# Chemical identification of an active component and putative neural mechanism for repellent effect of a native ant’s odor on invasive species

**DOI:** 10.3389/fphys.2022.844084

**Published:** 2022-08-30

**Authors:** Tatsuya Uebi, Tomoya Sakita, Ryo Ikeda, Keita Sakanishi, Tomoaki Tsutsumi, Zijian Zhang, Huiying Ma, Ryosuke Matsubara, Shigeru Matsuyama, Satoko Nakajima, Rong-Nan Huang, Shunya Habe, Abraham Hefetz, Mamiko Ozaki

**Affiliations:** ^1^ Department of Biology, Graduate School of Science, Kobe University, Kobe, Japan; ^2^ KYOUSEI Science Center for Life and Nature, Nara Women’s University, Nara, Japan; ^3^ Department of Chemistry, Graduate School of Science, Kobe University, Kobe, Japan; ^4^ Graduate School of Life and Environmental Sciences, University of Tsukuba, Tsukuba, Japan; ^5^ Graduate School of Life and Environmental Sciences, Kyoto Prefectural University, Kyoto, Japan; ^6^ Department of Entomology, National Taiwan University, Taipei, Taiwan; ^7^ School of Zoology, Tel Aviv University, Tel Aviv, Israel; ^8^ Department of Chemical Science and Engineering, Graduate School of Engineering, Kobe University, Kobe, Japan; ^9^ Morphogenetic Signaling Team, RIKEN Center for Biosystems Dynamics Research, Kobe, Japan

**Keywords:** invasive ant, cuticle hydrocarbon, repellent, olfactory response, brain activity

## Abstract

The invasive Argentine ants (*Linepithema humile*) and the red imported fire ants (*Solenopsis invicta*) constitute a worldwide threat, causing severe disruption to ecological systems and harming human welfare. In view of the limited success of current pest control measures, we propose here to employ repellents as means to mitigate the effect of these species. We demonstrate that cuticular hydrocarbons (CHCs) used as nestmate-recognition pheromone in the Japanese carpenter ant (*Camponotus japonicus*), and particularly its (*Z*)-9-tricosene component, induced vigorous olfactory response and intense aversion in these invasive species. (*Z*)-9-Tricosene, when given to their antennae, caused indiscriminate glomerular activation of antennal lobe (AL) regions, creating neural disarray and leading to aversive behavior. Considering the putative massive central neural effect, we suggest that the appropriate use of certain CHCs of native ants can facilitate aversive withdrawal of invasive ants.

## Introduction

The vast spread and high abundance of invasive species seriously threatens native ecosystems as well as affecting biodiversity conservation worldwide (Human and Gordon, 1996; Tsutsui et al., 2000; Suarez and Case, 2002; Powell et al., 2009; Gao and Reitz, 2017; Pyšek et al., 2017). For example, the Argentine ant (*Linepithema humile*), which is on the IUCN (International Union for Conservation of Nature) list of the 100 world’s worst invasive species, is one of the most widely-distributed invasive pests in six continents and the Pacific islands, including New Zealand, Hawaii, and Japan (Lowe et al., 2000; Giraud et al., 2002; Suhr et al., 2010; Van Wilgenburg et al., 2010; Ward et al., 2010; Mothapo and Wossler, 2011; Sato et al., 2017). Despite the extensive ongoing research aimed at the implementation of chemical control programs using pesticides or poison bait, only limited success has been achieved to date in both reducing population growth, and preventing new invasions (Silverman and Brightwell, 2008; Westermann et al., 2016). For example, in Tokyo, Japan, starting from very early stage of *L. humile* invasion, administration of insecticide had been carried out, and the pest was eradicated 38–42 months after. While in Kyoto, *L. humile* first invaded in 2008 have not completely been controlled even after 14 years of struggling (Inoue et al., 2015; Sakamoto et al., 2016; Sakamoto et al., 2017).

Although *L. humile* workers have a small body size (e.g., 2–2.5 mm), they can cooperate to attack and repel larger ant species such as the Japanese carpenter ant *Camponotus japonicus* (e.g., 7–14 mm), which often culminates in the death of the latter (Supplementary Movie S1). The promotion of cooperative behavior in many ant species’ societies is facilitated through the use of complex, species- and colony-specific cocktails of CHCs as chemical cues, designed to discriminate between friend and foe (Bonavita-Cougourdan et al., 1987; Ozaki et al., 2005; Ozaki et al., 2012; Ozaki and Hefetz, 2014). These cues also function to enable the ants to assess the relative dominance between individuals within the colony, and/or con- and heterospecific enemies or competitors (Briffa, 2010) which, accordingly, adjust their response, e.g., fight-or-flight. Hence, we predicted that the generally aggressive-offensive nature of *L. humile* would switch to retreat when exposed to large amounts of chemicals emitted from a hetero-specific ant that might be recognized as a dominant foe. As was expected, we found that *C. japonicus* CHCs (Ozaki et al., 2005), when highly concentrated, repelled *L. humile* in our chemical ecological study of ant.

In the present study, we succeeded to discover the most effective repellent among CHCs of *C. japonicus*, using artificially synthesized all its CHC components. In order to step into the underlying neurophysiological mechanism, we investigated the (*Z*)-9-tricosene, one of the most aversive CHCs. However, because of the body size problem, it was impossible to conduct anterograde staining or electrophysiological recording from a single CHC-sensillum called sensillum basiconicum in *L. humil*e but a large ant species, *C. japonicus*. We also examined (*Z*)-9-tricosene-induced glomerular activation of the worker brain in both *L. humile* and *C. japonicus*, using anti-pERK antibody-staining method (Yabuki et al., 2016; Maeda et al., 2020). Based on those experimental results and neurological background of the behavior of worker ants, which have been accumulated many in *Camponotus* species (Zube et al., 2008; Zube and Röessler, 2008; Nakanishi et al., 2010; Nishikawa et al., 2012), we estimated the mechanism for the aversive behavior of *L. humile* workers toward (*Z*)-9-tricosene.

In addition, we found that (*Z*)-9-tricosene was effective on several ant species including *L. humile* and *Solenopsis invicta* in a dose-dependent manner. Indicating the field experimental data on a barrier effect of (*Z*)-9-tricosene, we propose a safe and strong repellent here for the deterrence and/or containment of such an invasive pest.

## Materials and methods

### Ants

Argentine ant (*Linepithema humile*) workers were collected from several sites in Kobe, Japan, and kept in the laboratory in the plastic boxes (26 × 35 × 6 cm^3^ or 22 × 16 × 9 cm^3^), lined with Fluon powder in order to prevent the ants from escaping. A glass tube (12.5 cm length, 1.5 cm diameter) covered with a red transparent acrylic sheet served as nesting artificial ant nest, and equipped with a wet cotton wool for providing humidity. The ants were fed either with chopped mealworms as a protein-rich food source and 30% diluted honey, weekly, or with Bhatkar-Whitcomb diet and 0.1 M sucrose twice a week. Workers of *Formica japonica* and *Camponotus japonicus* were collected at the campus of Kobe University, and *Monomorium pharaonis* and *Pristomyrmex punctatus* workers were donated by Earth Corporation, Tokyo Japan. They were reared and fed as described above for the Argentine ants. Collections and assay with the red imported fire ants, *Solenopsis invicta*, were done in Taoyuan, Taiwan, and Taiwan University.

### Purification of cuticular hydrocarbons of *C. japonicus*

CHC of *C. japonicus* were extracted by placing fully anesthetized workers (to prevent discharge of poison and Dufour’s gland constituents) in a 10-ml Spitz tube kept on ice and extracted with 2 ml *n*-hexane for 5 min. The body-wash solution from 5 workers was concentrated in an evaporator up to an approximate volume of 2 ml, and subsequently purified by a silica gel column. The silica gel column was first washed with 2 ml dichloromethane under gentle air pressure and then equilibrated with 4 ml *n*-hexane. The applied CHC extract was then eluted using 3 ml *n*-hexane. Aliquots of the eluted CHC were stored at −20°C until just before use.

### Preparation of hydrocarbons

Twenty-four synthetic hydrocarbons were used in this study. Six *n*-alkanes ranging from *n*-tricosane to *n*-nonacosane, and one alkene: (*Z*)-9-tricosene, were commercially purchased (Tokyo Chemical Industry Co., Ltd., Tokyo, Japan). Nine alkenes ranging from (*Z*)-7-tricosene to (*Z*)-7-nonacosene, and (*Z*)-9-pentacosene to (*Z*)-9-nonacosene; 8 methyl-branched alkanes: (*S*)-5-methylheptacosane, (*R*)-5-methylheptacosane, (*S*)-13- methylheptacosane, (*R*)-13-methylheptacosane, 7,15-dimethylheptacosane, 5,7,12-trimethylheptacosane, 7,9,12-trimethylheptacosane and 5,7,12-trimethylpentacosane were chemically synthesized by us (See below and Supplementary Text for details of the chemical synthesis).

### Chemical synthesis of cuticular hydrocarbons

All starting materials for hydrocarbon syntheses were obtained from commercial sources or synthesized using standard procedures. Reactions were conducted in well-cleaned glassware with magnetic stirring under an atmosphere of dry argon using Schlenk and vacuum techniques. For intermediate and final product checking, 1H and 13C NMR spectra (400 and 100 MHz, respectively) were recorded on a Bruker Avance III HD 400 using TMS (0 ppm) and CDCl3 (77.0 ppm) as the internal standards, respectively. Mass spectra were measured using a Thermo Finnigan LCQ TRACE GC ULTRA (EI). Preparative column chromatography was performed using Kanto Chemical silica gel 60 N (spherical, neutral), Fuji Silysia BW-4:10MH silica gel or YMC_GEL Silica (6 nm I-40–63 μm). Thin layer chromatography (TLC) was performed using Merck 25 TLC silica gel 60 F254 aluminum sheets. We discriminately synthesized both enantiomers for the two monomethyl-branched alkanes, and plausible stereoisomers for the dimethyl- and trimethyl-branched alkanes, the 5 stereochemistry of which was putatively assigned according to the biosynthesis of the ant CHCs (Evans et al., 1982; Wang et al., 1984; Blomquist et al., 2010; Belloa et al., 2015). The chirality of methyl substituents was introduced via a chiral pool method using enantiopure citronellols, asymmetric alkylation with Evans chiral auxiliary and enzymatic desymmetrization of a meso diol. Further details of the chemical synthesis are presented 4 “Supplementary Text and its References”.

### Gas-chromatography

CHCs of *C. japonicus* were analyzed by Gas-chromatography (GC) using a DB-1HT non-polar capillary column (15 m length, 0.25 mm i.d. and 0.1 μm film thickness) and a flame ionization detector. The injector and detector temperatures were both set at 300°C. The oven temperature was held at 60°C for 1 min, increased at 10°C/min to 300°C, with a final hold of 5 min. We used helium as the carrier gas. We injected 1 μl *n*-hexane containing 0.05-ant-equivalent CHCs. By comparing our data with previously reported data (Ozaki et al., 2005), we confirmed good reproducibility of the CHC purification process.

### Behavioral assay for cuticular hydrocarbon examination

Behavioral assays were conducted in a round arena (6 cm diameter), placed in the ants’ rearing box. Each assay comprised several workers exposed to the test solution, which was applied on a glass stick having a bead-shape spherical head (2 mm diameter). Test solution comprised serial dilutions of either whole CHC extract or CHC fractions dissolved in *n-*hexane, starting from 10-ant-equivalent dose/10 μL, or each of the 24 synthetic hydrocarbons, starting from 50 μg/10 μL. At the onset of each assay, the test solution was applied to the spherical bead that after allowing complete evaporation of the hexane solvent was gently held close to the test ant until the ant spontaneously touched the bead. The ant behavior was immediately recorded as either ignore, avoidance, or aversive behaviors. Only ants that ignored the *n*-hexane-treated glass bead beforehand were used for the behavioral assays. A total of 10 ants were tested for each dose of ant CHC extracts, their fraction, or individual synthetic hydrocarbons. Each ant was exposed five times to the same amounts of each sample at 5-min-intervals, and the average frequencies of ignore, avoidance and aversive behavior were calculated.

### Histochemical fluorescent staining using anti-pERK antibody

The workers of *L. humile* were anesthetized on ice for about 5 min, immobilized between small plastic sponges, and allowed to reach room temperature. Both antennae of each ant were contacted to a glass bead (2 mm diameter) treated either with *n*-hexane (control) or 50 μg (*Z*)-9-tricosene dissolved in *n*-hexane for 4 min, after which the ants were anesthetized on ice for 1 min in order to dissect their heads. The heads were immediately fixed in 4% paraformaldehyde in 0.1 M phosphate buffer overnight at 4°C. After fixation, the heads were subsequently incubated in Ringer’s solution (4.8 mM TES, 127 mM NaCl, 6.7 mM KCl, 2 mM CaCl_2_, and 3.5 mM sucrose) containing first 10% and then 20% sucrose for 1 h at 4°C in the same Ringer’s solution, followed by 30% sucrose in the same Ringer’s solution overnight at 4°C. The heads were embedded in O.C.T. compound (Sakura Finetek Japan Co., Ltd., Tokyo, Japan) and frozen in a cryostat (CM1850; Leica Biosystems Nussloch GmbH, Wetzlar, Germany) to make 10 μm-thick vertical sections. The frozen head sections were mounted on glass slides, dried for 1 h and washed three times, for 5 min each, in phosphate-buffered saline containing 0.1% Triton-X (PBST). It was subsequently activated by incubation in HistoVT One (NACALAI TESQUE, INC., Kyoto, Japan) for 20 min at 70°C, followed by three washes with PBST. The slides were then treated with Blocking One (NACALAI TESQUE, INC., Kyoto, Japan) for 2 h at room temperature and incubated with anti-pERK antibody (phospho-p44/42 MAPK (Erk1/2) (Thr202/Tyr204) (D13.14.4E) XP Rabbit mAb #4370; Cell Signaling Technology Inc., Danvers, MA, United States) at 1:500 dilution with Can Get Signal solution A (NKB-501; TOYOBO CO., LTD., Osaka, Japan) overnight at 4°C. On the next day, the sections were washed four times in PBS for 5 min each and incubated with anti-sypapsin antibody (3C11; The Developmental Studies Hybridoma Bank, the University of Iowa, Iowa, IA, United States) at 1:50 dilution with Can Get Signal solution B (NKB-601; TOYOBO CO., LTD., Osaka, Japan) overnight at 4°C. On the following day, the sections were washed four times in PBS for 5 min each and incubated with Alexa 594-conjugated goat anti-rabbit IgG secondary antibody (1:500 diluted; A11012; Thermo Fisher Scientific, Wilmington, DE, United States) plus Alexa 405-conjugated goat anti-mouse IgG secondary antibody (1:500 diluted; A31553; Thermo Fisher Scientific, Wilmington, DE, United States) diluted with Can Get Signal solution B overnight at 4°C. Subsequently the slides were washed four times in PBST for 5 min each and mounted with Fluoromount (K024; DIAGNOSTIC BIOSYSTEMS, Pleasanton, CA, United States). Finally, we acquired the fluorescence images using a confocal microscope (Model FV1000, Olympus Corporation, Tokyo, Japan) coupled with a fluorescent microscope (Model BX61W1, Olympus Corporation, Tokyo, Japan) with a 20× objective lens (UPLSAPO, NA0.75; Olympus Corporation, Tokyo, Japan). The excitation wavelength for pERK-staining images was 559 nm while emission was collected from 614 to 624 nm. For synapsin staining-images, the excitation wavelength was 405 nm, and emission was collected from 418 to 428 nm.

When we used *C. japonicus* instead of *L. humile*, the workers were immobilized in disposable pipette tips with the top cut to fit the head of the ant, so that the head of the ant stuck out of the opening. The antennae were stimulated for 4 min by contact with a glass stick having a bead-shape spherical head loaded *n*-hexane (control) or 50 μg or 1000 μg (*Z*)-9-tricosene dissolved in *n*-hexane. For the positive control experiment, the antennae were set about 1 cm far from an Eppendorf tube filled with or without benzaldehyde (Tokyo Chemical Industry Co., Ltd., Tokyo, Japan). Then the antennae were exposed to the vapor of benzaldehyde for 5 min.

After such a contact or vapor stimulation, the ants were anesthetized on ice for 1 min in order to dissect their heads. The brains were dissected and immediately fixed in 4% paraformaldehyde in 0.1 M phosphate buffer overnight at 4°C. After fixation, they were subsequently activated by incubation in HistoVT One for 45 min at 90°C, followed by three washes with PBST. The brains were then treated with Blocking One (NACALAI TESQUE, INC., Kyoto, Japan) for 2 h at room temperature and incubated with anti-pERK antibody at 1:500 dilution with Can Get Signal solution A overnight at 4°C. On the next day, the brains were washed four times in PBS for 5 min each and incubated with anti-synapsin antibody at 1:50 dilution with Can Get Signal solution B overnight at 4°C. On the following day, the brains were washed four times in PBS for 5 min each and incubated with Alexa 594-conjugated goat anti-rabbit IgG secondary antibody plus Alexa 405-conjugated goat anti-mouse IgG secondary antibody diluted with Can Get Signal solution B overnight at 4°C. Subsequently, they were washed three times in PBST and dehydrated by incubation in an ethanol series (70%, 80%, 90%, 95%, 100%, and 100%), and penetrated with methyl salicylate, finally. The brains were stored in the dark at 4°C until microscopic observation. We acquired the fluorescence images using a confocal microscope (C2, Nikon Corporation, Tokyo, Japan) coupled with a fluorescent microscope Ti2-E, Nikon Corporation, Tokyo, Japan) with a 20× objective lens (plan Apo VC, NA0.75; Nikon Corporation, Tokyo, Japan). The excitation wavelength for pERK-staining images was 543 nm while emission was collected 585 nm. For synapsin staining-images, the excitation wavelength was 405 nm, and emission was collected from 447 nm. Fluorescence images of optical sections (2 μm thickness) were analyzed with NIS elements AR software ver. 5.01 (Nikon Corporation, Tokyo, Japan).

### Brightness evaluation of the fluorescent signal

In order to discriminate between non-activated and activated areas in the ant brain, the brightness of a specified glomerulus or cluster of glomeruli was compared between non-stimulated and stimulated conditions. When we focus on a specific glomerulus concerned, its brightness was normalized to that of an adjacent non-activated glomerulus in the non-stimulated and stimulated samples, and thus the normalized values of brightness were compared with each other. When we compare the brightness of whole T6 region, its average brightness per unit area was normalized to that of the adjacent non-activated area non-stimulated and stimulated samples, and those normalized average values were compared with each other. Statistical significance test was done by Mann-Whitney’s U test. Brightness measuring and comparative calculation were conducted, using NIS elements AR software ver. 5.01 (Nikon Corporation, Tokyo, Japan).

### Anterograde staining with a fluorescent dye

A worker ant of *C. japonicus* immobilized on a slide glass using bee wax was set under an inverted microscope (CKX53; Olympus, Tokyo, Japan). The single s. basiconicum on an antenna was cut by hand with a small piece of razor blade. After a quick wash of the lesion in the middle of the sensillar shaft with distilled water, the antenna was immersed in 1% dextran-tetramethylrhodamine dissolved in distilled water and covered with a small piece of plastic wrap. The ants on a slide glass were then placed in a wet chamber and kept in the dark for 72 h at 4°C. Its brain was removed in phosphate-buffered saline (PBS; 128 mM NaCl, 5 mM KCl, 2 mM MgCl_2_, 1 mM Na_2_HPO_4_, 0.34 mM KH_2_PO_4_, 1.83 mM CaCl_2_, and 25 mM glucose), fixed in 4% paraformaldehyde in PBS overnight at 4°C, washed with PBS, and dehydrated in an ethanol series consisting of 50%, 70%, 80%, 90%, 95%, and 100% (twice) each for 10 min. Finally, the brains were soaked in methyl salicylate and observed under a confocal laser scanning microscope (A1RMP and A1R DU4-GaAsP; Nikon Corporation, Tokyo, Japan).

Whole-mount brain specimens of 8 ants, in which the fluorescent dye was introduced into the brain, were imaged with a confocal laser scanning microscope (Zeiss LSM510, Carl Zeiss AG, Jena, Germany) using a Plan-Neofluar 10×, 20×, or 40× objective (numerical aperture, 0.3, 0.5 or 0.75, respectively). Serial optical slices were acquired from posterior to anterior at approximately 1.0-μm intervals. For the reconstruction of axonal projection, a confocal stack of approximately 125 optical sections was analyzed using Amira software (Indeed Visual Concepts GmbH, TGS Inc., Berlin, Germany). In each optical section, we demarcated each glomerulus and the basiconic sensillar olfactory receptor neurons (ORNs), and drew the contours by hand using a Wacom LCD tablet (PL550, Wacom Co., Ltd., Saitama, Japan).

In accordance with the method of Nakanishi et al. (Nakanishi et al., 2010), we constructed the glomerular map to be referred for identification of the axonal projection region in an AL from the s. basiconicum or specified glomeruli by pERK staining.

### Tip-recording with a thin film of hydrocarbon-containing oil

The head of anaesthetized *C. japonicus* worker was dissected and set under a microscope (Model BX53, Olympus, Tokyo, Japan) in an electrophysiological setup. The indifferent electrode was inserted into the head at the base of the antenna. The glass capillary (Model G-1, Narishige, Tokyo, Japan) serving as a stimulating and recording electrode was pulled using a laser puller (Model P-2000, Sutter Instrument Co., CA, United States), so that the tip opening diameter was slightly wider than that of the s. basiconica. The electrode was filled with 10 mM NaCl and overlaid with a 3-μm-thick film of silicon oil containing 0%, 1%, 10% or 100% (*Z*)-9-tricosene at the very tip. For single sensillar stimulation, the electrode was manipulated by the 3D hydraulic micromanipulator (Model MMO-203, Narishige, Tokyo, Japan), to engulf the tip of a s. basiconicum with the NaCl solution through the (*Z*)-9-tricosene-containing silicon oil film. The impulse response to the contact stimulation with 0, 1, 10 or 100% (*Z*)-9-tricosene was serially recorded for 6 s by a data acquisition controller system, IDAC4 (Ockenfels Syntech GmbH, Buchenbach, Germany) with AutoSpike32 software (Ockenfels Syntech GmbH, Buchenbach, Germany). The interval between stimuli was longer than 10 min. The number of tested sensilla were 13, 5, 10, 18 for the stimulation with 0%, 1%, 10% and 100% (*Z*)-9-tricosene, respectively. In this experiment, we used one sensillum per ant.

### Field trap test using repellent barrier

Two percent of (*Z*)-9-tricosene diluted in *n*-hexane (for test) or plane *n*-hexane (for control) was sprayed around the box type polypropylene trap (8.8 × 19.5 × 2.2 cm^3^) with a sticky surface (8 × 8 cm^2^) (Semco Co, Ltd. Hiroshima, Japan) put on a plastic plate (12 × 12 cm^2^), so that the box trap was surrounded by the (*Z*)-9-tricosene barrier. The amount of (*Z*)-9-tricosene used per plate was calculated to be 0.1 or 1 mg/cm^2^. At each dose, 6 pairs of test and control traps were prepared without any attractants and set at 6 observation points in the outdoor field (around the Port Island campus of RIKEN, Kobe, Japan), where *L. humile* were usually observed. Those observation points were more than 5 m separated from each other, and at each observation point the test and control traps were set next to each other and left for 2 weeks. We counted the number of *L. humile* in the test and control traps 3 times (day 3, day 7 or 10, and day 14) and the significant difference examination was done by Mann-Whitney’s U test between the test and control traps in each pair.

## Results

### Behaviourally effective repellent toward *L. humile*

**FIGURE 1:**
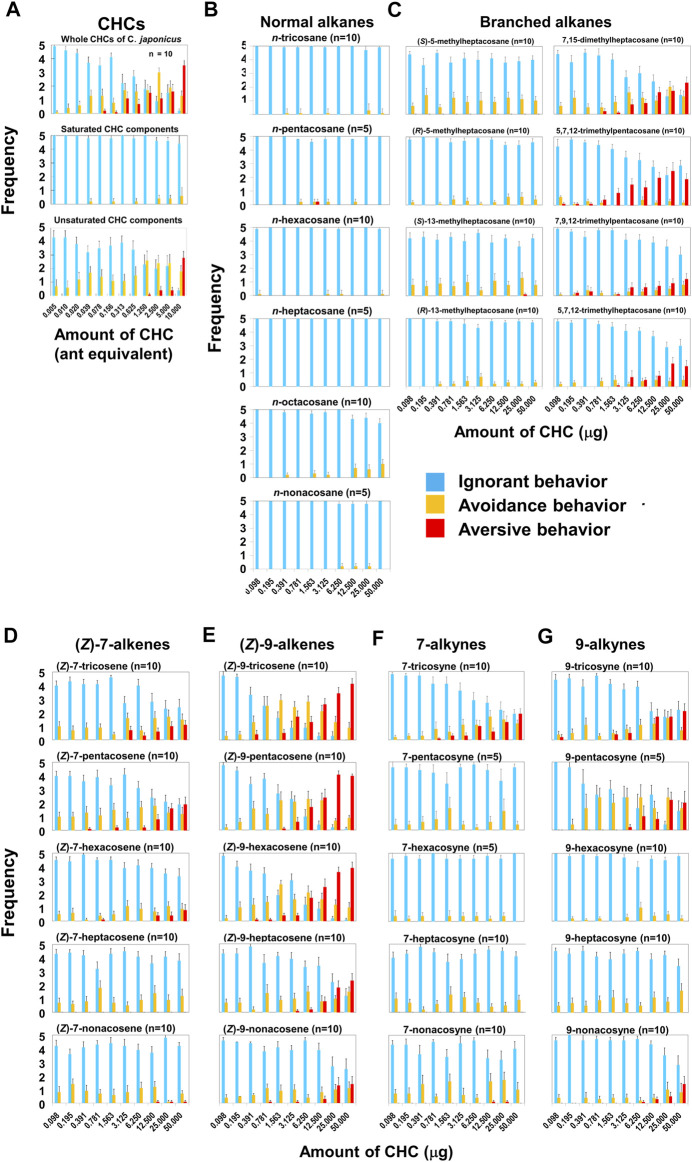
Behavioral effects of *C. japonicus* cuticular hydrocarbons (CHCs) and related synthetic compounds on *L. humile*. **(A)** Behavioral effects of whole *C. japonicus* CHC extracts (Top), and purified saturated (Middle) and unsaturated (Bottom) fractions. **(B**–**E)** Behavioral effects of normal alkanes, branched alkanes, (*Z*)-7-alkenes and (*Z*)-9-alkenes, respectively. **(F,G)** Behavioral effects of 7-alkynes and 9-alkynes, respectively. In Figures 1, 4, 9, blue, yellow, and red columns indicate the average frequencies of ignore, avoidance and aversive behaviors with standard errors, respectively.

**FIGURE 2:**
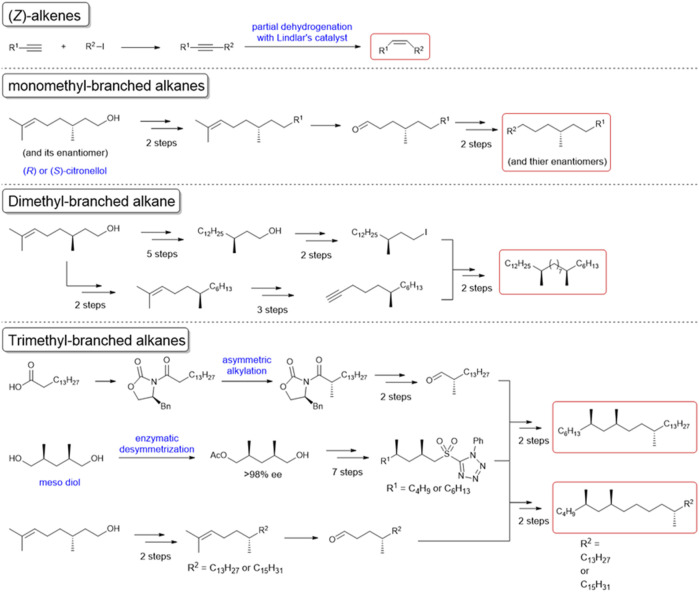
Chemical synthesis of the cuticular hydrocarbon components of *C. japonicus*. The schemes of the syntheses of unsaturated or methyl branched components are shown. The starting materials and reagents for each synthesis are readily available from commercial sources.

In our behavioral experiments, *L. humile* workers were exposed to glass beads (2 mm diameter) coated with various amounts of *C. japonicus* CHCs, at doses of up to 10-ant-equivalents or up to 50 μg of synthetic CHC components (Supplementary Materials). Figure 1A presents the reaction of worker *L. humile* to *C. Japonicus* whole CHC extract, classifying three behaviors: ignore (blue), avoidance (yellow), and aversion (red) (see Supplementary Movies S3−S5). At lower doses the *L. humile* workers’ response was either to ignore or, at most, simple avoidance. However, at doses higher than 2.5-ant-equivalents, worker behavior became aversive, characterized by rapid withdrawal following intensive self-grooming of the antennae (Supplementary Movie S2). The frequency of aversion increased with the increase in CHC dose (Figure 1A Top). To identify the key components in *C. japonicus* CHCs that trigger this behavior, we fractionated the extract into saturated and unsaturated components using AgNO_3_ column chromatography. While the saturated CHC fraction did not lead to any behavioral response, the unsaturated CHC fraction induced intense aversive behavior at high doses, comparable to that obtained using whole extracts (Figure 1A Middle and Bottom). Next, we tested a suite of synthetic hydrocarbons present in *C. japonicus* CHC extract (Evans et al., 1982; Wang et al., 1984; Blomquist et al., 2010; Belloa et al., 2015). (*Z*)-alkenes (which are unavailable commercially) were synthesized from the corresponding alkynes via partial hydrogenation by Lindlar’s catalyst (Figure 2). We thereby examined every CHC component of *C. japonicus*, in regard to its stereochemistry. None of the normal alkanes induced either avoidance or aversive behaviors in *L. humile*, except for octacosane, which at high doses induced avoidance behavior (Figure 1B). Among the branched alkanes, the *S* enantiomers of 5- and 13-methylheptacosane induced moderate avoidance behavior, while the *R* enantiomers induced only a very weak response. In contrast, the dimethyl and trimethyl-branched alkanes induced a rather strong aversive behavior (Figure 1C). Specifically, 7, 15-dimethylheptacosane and 5, 7, 12-trimethylpentacosane elicited significantly more intense aversive responses than those elicited by 7, 9, 12-trimethylpentacosane and 5, 7, 12-trimethylheptacosane (*p* < 0.05, Mann-Whitney’s U test). The ignore response was negatively correlated with the amount of CHCs (r = −0.5192 to −0.3954, *p* < 0.01, Pearson’s correlation coefficient test), while the aversive response was positively correlated (r = 0.3776 to 0.5112, *p* < 0.01, Pearson’s correlation coefficient test).

Among the (*Z*)-7-alkenes, (*Z*)-7-pentacosene was most effective (*p* < 0.05, Mann-Whitney’s U test) in inducing an aversive response (Figure 1D), while (*Z*)-7-tricosene and hexacosene had a lesser effect. The (*Z*)-7-nonacosene elicited only a minute response at high doses, while (*Z*)-7-heptacosene did not elicit any activity. In contrast, all the (*Z*)-9-alkenes elicited a response that increased in frequency in a dose-dependent manner (Figure 1E, r = 0.480 to 0.808, *p* < 0.01, Pearson’s correlation coefficient test), and the magnitude of which was also higher than the ants’ responses to the (*Z*)-7-alkenes. Avoidance behavior was apparent at all doses, while aversion behavior occurred mostly at high doses. (*Z*)-9-tricosene, (*Z*)-9-pentacosene, and (*Z*)-9-hexacosene, which are minor components of *C. japonicus* CHCs (Ozaki et al., 2005), were remarkably effective, inducing higher frequencies of aversive behavior than both (*Z*)-9-heptacosene and (*Z*)-9-nonacosene (*p* < 0.05, Mann-Whitney’s U test).

### Antennal lobe activation by the repellent

**FIGURE 3:**
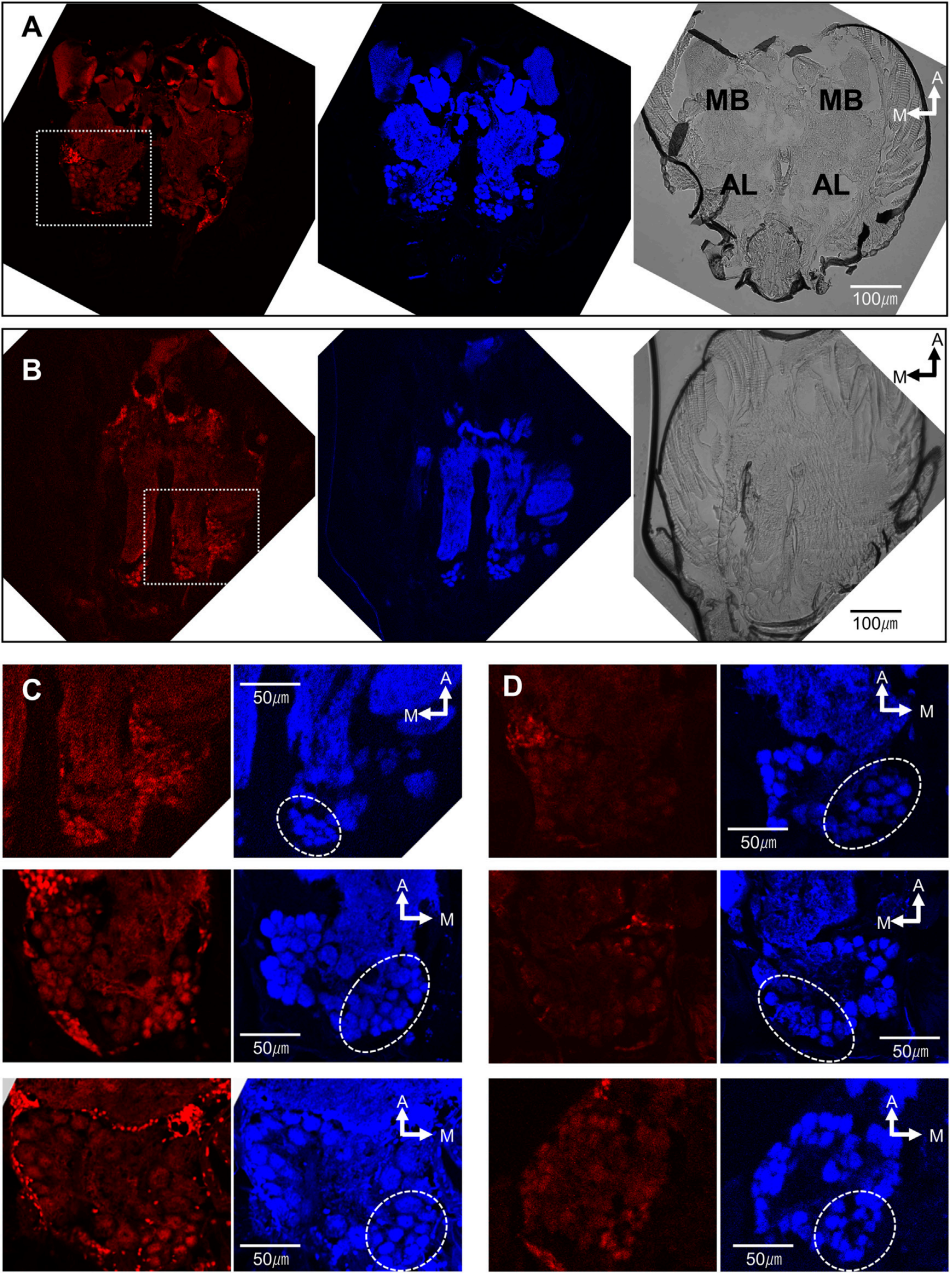
The brain of *L. humile* activated by the contact stimulation of antennae with (*Z*)-9-tricosene. **(A−D)** (Left) Histochemical fluorescent staining with anti-pERK-antibody; (**A,B)** (Middle); **(C,D)** (Right) Histochemical fluorescent staining with anti-synapsin-antibody, corresponding to **(A−D)** (Left), respectively; **(A,B)** (Right) Nomarski differential interference microscopic images, corresponding to **(A,B)** (Left), respectively. MB, Mushroom body. AL, antennal lobe. **(A)** The representative brain of the ant, chemically non-stimulated. **(B)** The representative brain of the ant, chemically stimulated with (*Z*)-9-tricocene. **(C)** (Top) Magnified image of the dotted-line square area in **(B)** (Left). **(D)** (Top) Magnified image in the same brain as **(A)** (Left). **(C)** (Middle and Bottom) Magnified images in two other brains at the same level but in the opposite side AL of **(C)** (Top). Dotted circles indicate the glomerular clusters of T6-like region on the anti-synapsin-antibody staining images.

The high effectiveness of (*Z*)-9-tricosene and its commercial availability made it an excellent candidate for further probing the possible neural mechanism that leads to the aversion response. Consequently, we stimulated the antennae of *L. humile* with (*Z*)-9- tricosene and identified which brain regions were activated, using histochemical fluorescent staining with anti-pERK antibody (Yabuki et al., 2016; Maeda et al., 2020) (see Supplementary Materials for details). A 4-minute stimulation with a control, clean glass bead, did not evoked any specific staining (Figure 3A Left), whereas glass beads coated with 50 μg (*Z*)-9-tricosene, constituting a large enough amount to induce aversive behavior (see Figure 1E), resulted in indiscriminate staining of multiple glomeruli in the AL, the primary olfactory center in insects (Figures 3B,C Left). Although the control and test were carried out on different individuals, the brain sections in both are at the same level of optical section along the dorsoventral axis. Figure 3C Top presents a magnified image of the AL area of Figure 3B Left (delineated by a dotted-line square). Two more images of the AL are seen in Figures 3C Middle and Bottom, representing sections at mostly the same level. Figures 3A,B Middle present the histochemical fluorescent staining for whole neural tissues with anti-synapsin antibody corresponding to Figures 3A,B Left, respectively. Figures 3A,B Right are the Nomarski differential interference microscopic images corresponding to Figures 3A,B Left, respectively. In total, we observed five control slices and nine test slices randomly made from the fixed specimen of at least 15 ant brains, respectively. Focusing on the posterior-median region surrounded by a dotted circle in Figures 3C,D, the control slices were not clearly stained in any glomeruli by the anti-pERK-antibody method (see Figure 3D), while the test slices demonstrated multiple glomeruli stained as shown in Figure 3B left or Figure 3C Left.

**FIGURE 4:**
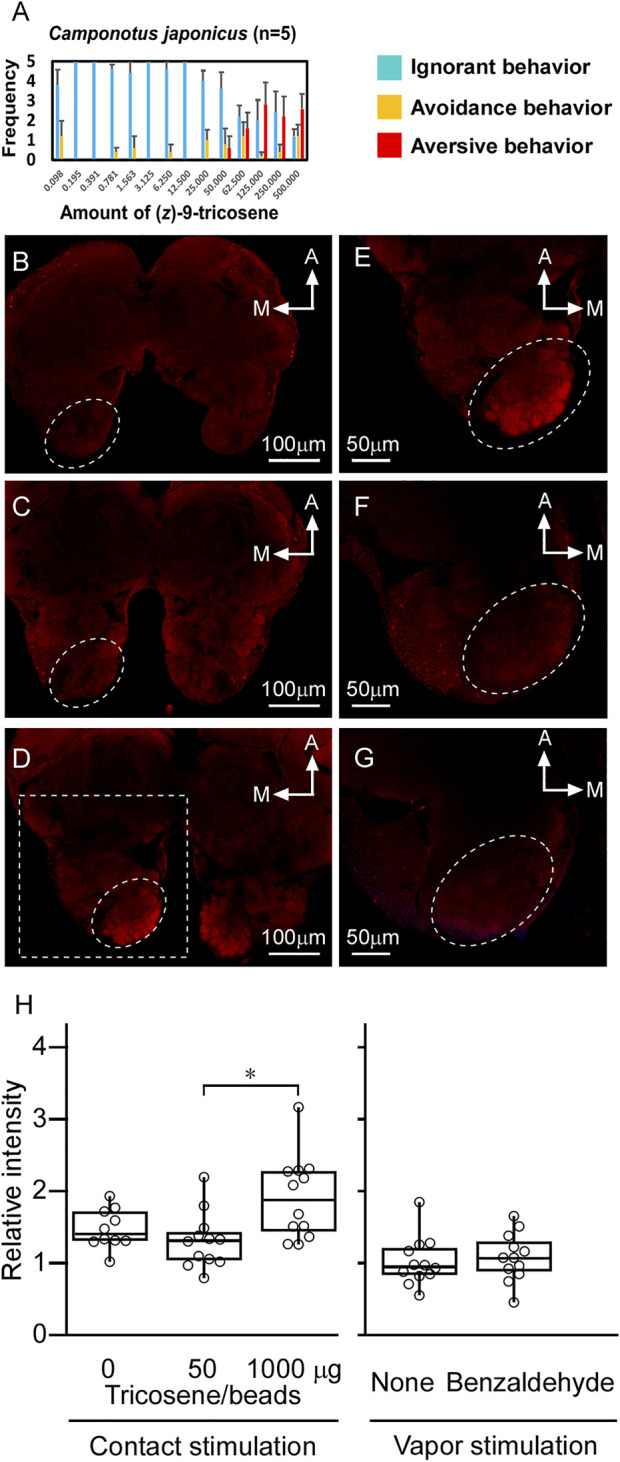
The brain of *C. japonicus* activated by the contact stimulation of antennae with (*Z*)-9-tricosene. **(A)** Dose-dependent behavioral effect of (*Z*)-9-tricosene on *C. japonicus*, including at larger amounts than 50μg/bead (*Z*)-9-tricosene. **(B−D)** The representative set of histochemical fluorescent images of anti-pERK-antibody staining, when the antennae were stimulated with 0, 50, and 1000μg/bead of (*Z*)-9-tricosene, respectively. **(E)** Magnified image of the dotted-line square area in **(D)**. As a positive control, the antennae were stimulated with benzaldehyde vapor. **(F,G)** The representative optical slices at the level of T6 region under the non-stimulated and stimulated conditions, respectively. Dotted circles indicate the glomerular clusters of T6 region. **(H)** Comparison in brightness of the identified glomerulus between non-stimulated and stimulated conditions. Asterisk indicates significant difference (*p* < 0.05, Mann-Whitney’s U test).

In 3.6, we mention that the workers of *C. japonicus* were insensitive to the smaller amounts of (*Z*)-9-tricosene than 50 μg/glass bead (see Figure 8F). However, they did avoid larger amounts of (*Z*)-9-tricosene than that in a dose-dependent manner (Figure 4A). Thus, we conducted the same histochemical experiment also in the brain of *C. japonicus*, when (*Z*)-9-tricosene were contacted to their antennae at 0, 50, and 1000 μg/glass bead, respectively (Figure 4B−4D). Figure 4E presents a magnified image of the AL area of Figure 4D (delineated by a dotted-line square). It was then found that the antennae stimulation only with 1000 μg/bead of (*Z*)-9-tricosene evoked clear activation in a limited AL region corresponding to the T6 (see Figure 6A). As a positive control, the same experiment was performed, but using the vapor stimulation with benzaldehyde instead of the contact stimulation with (*Z*)-9-tricosene. Figures 4F,G show the representative AL images at the level of T6 region under the non-stimulated and stimulated conditions, respectively. In either case, obvious activation was not observed. Thus, the activation of the T6 glomeruli was significantly examined by statistical comparison in the brightness among AL images, when the antennae were stimulated with 0, 50, or 1000 μg/bead of (*Z*)-9-tricosene. Significant difference is indicated by an asterisk in Figure 4H Left. It suggests that the contact stimulation of antennae with 1000 mg/bead of (*Z*)-9-tricosene evoked significantly stronger activation of T6 than that with 50 mg/bead of (*Z*)-9-tricosene (*n* = 12, Examined T6 regions were in 12 ALs of 6 brains; *p* = 0.007, Mann-Whitney’s U test), which has no significant difference with the control level (*n* = 10, Examined T6 regions were in 10 ALs of 5 brains; *p* = 0.093, Mann-Whitney’s U test). However, there was no significant difference in the brightness between contact stimulations with 0 and 1000 mg/bead (*Z*)-9-tricosene (*p* = 0.093, Mann-Whitney’s U test). Also on the positive control, the activation of the T6 glomeruli was significantly examined by statistical comparison in the brightness between non-stimulated and stimulated conditions, and there was no significant difference (Figure 4H) (*p* = 0.59, Mann-Whitney’s U test).

**FIGURE 5:**
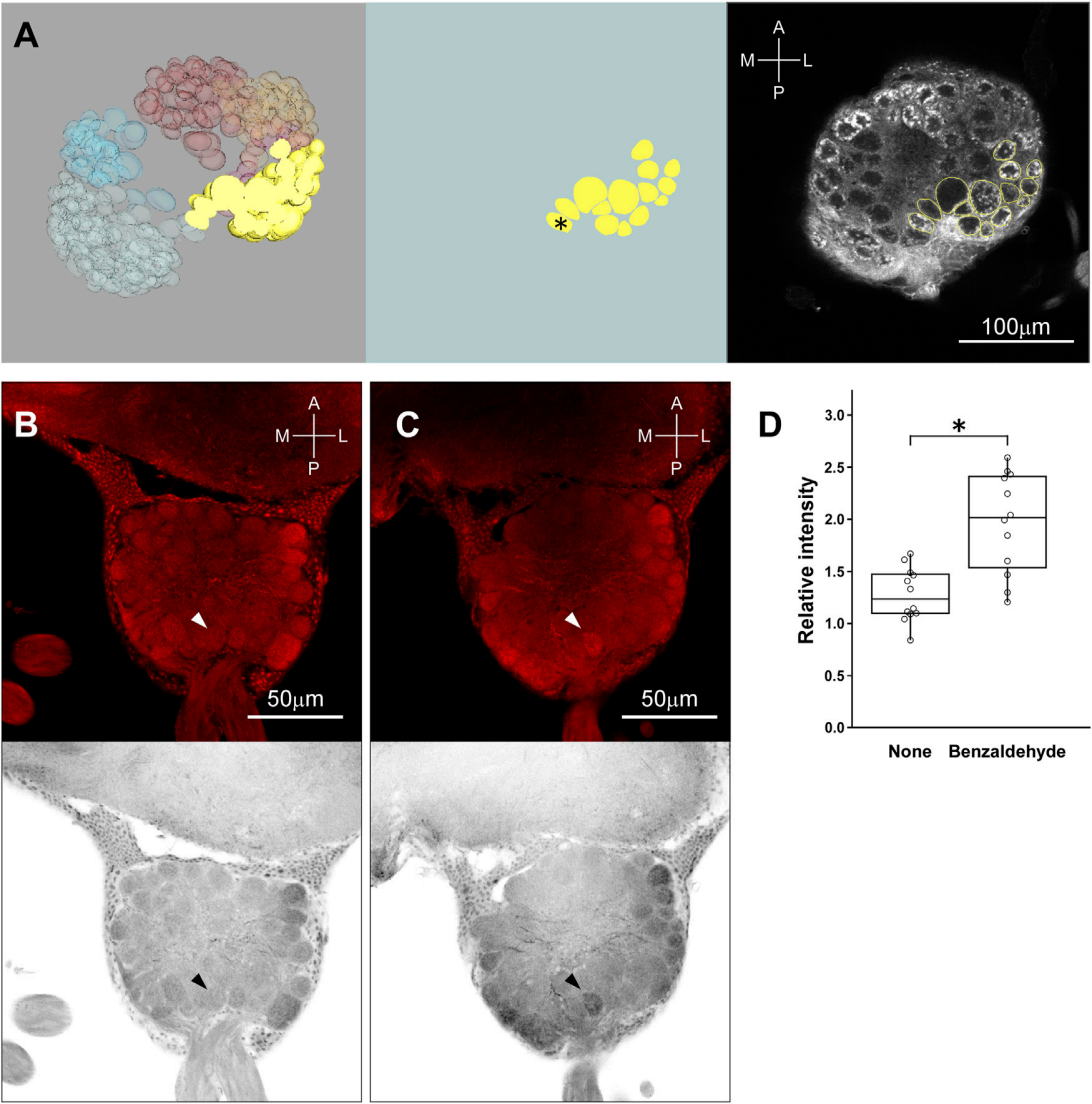
Identification of glomerulus activated by the stimulation with benzaldehyde vapor. **(A)** (Left) A frontal section of T2 is implanted in the dorsal part of reconstructed 3D glomerular model. **(A)** (Middle) A part of T2 glomeruli is extracted to identify the activated one. Asterisk indicates the candidate glomerulus activated by the vapor stimulation with benzaldehyde. **(A)** (Right) The contoured glomeruli of **(A)** (middle) were superimposed on the confocal image. **(B)** (Top, Bottom) The original image of AL at the level of T2 region under non-stimulated condition and its inverted gray scale image are indicated, respectively. **(C)** (Top, Bottom) The representative original image of AL at the level of T2 region with the glomerulus activated by the vapor stimulation with benzaldehyde. Arrowheads indicate the location of the glomerulus activated by the vapor stimulation with benzaldehyde. **(D)** Comparison in brightness of the identified glomerulus between non-stimulated and stimulated conditions. Asterisk indicates significant difference (*p* < 0.05, Mann-Whitney’s U test).

Benzaldehyde vapor was then activated a single glomerulus in T2 region. Referring to Figure 5A, which illustrates glomerular model construction processes at the level of T2 (Left, Frontal section was implanted in the dorsal part of reconstructed 3D glomerular model; Middle, Identified glomeruli in the T2 region are extracted; Right, Identified glomeruli were superimposed on the confocal image), the activated glomerulus in Figure 5C (arrowhead) was specified as that indicated by an asterisk in Figure 5A Middle. This glomerulus was not activated without vapor stimulation with benzaldehyde (Figure 5B, arrowhead). Brightness of activated glomeruli by benzaldehyde vapor (*n* = 12; Examined glomeruli were in 12 ALs of 6 brains) was compared with that without stimulation (*n* = 12; Examined glomeruli were in 12 ALs of 6 brains), and significant difference was statistically confirmed (*p* = 0.0009, Mann-Whitney’s U test) (Figure 5D).

### Axonal projection of the single sensillar olfactory receptor neurons

**FIGURE 6:**
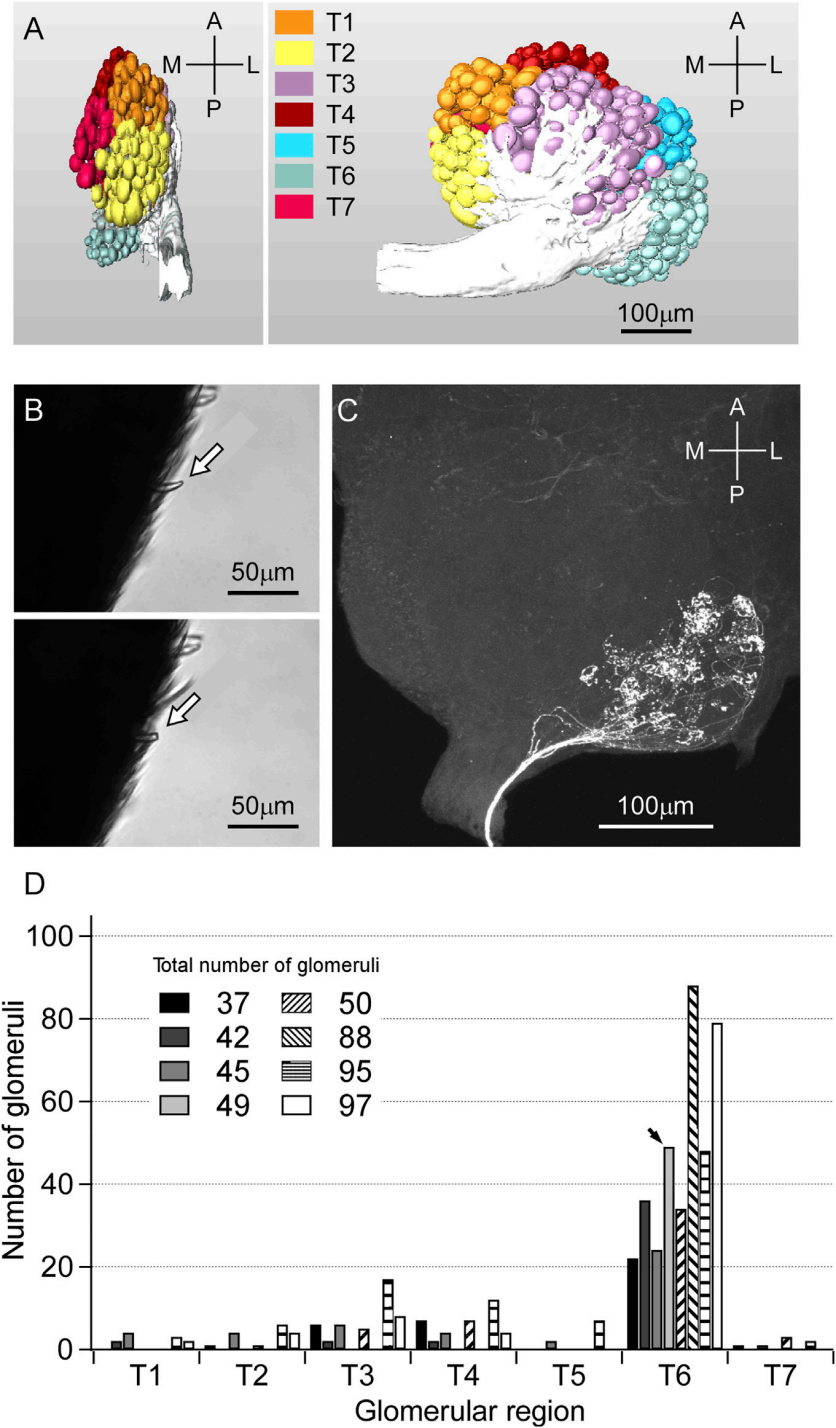
Glomerular map of *C. japonicus* and anterograde staining of the ORNs from a single s. basiconicum to AL. **(A)** (Left) Side view of the glomerular map. **(A)** (Right) Front view of the glomerular map. T1**−**T7 regions of AL are differently colored; anterior-lateral (orange), lateral (yellow), anterior-ventral (purple), dorsal (brown), dorsomedian (blue), posterior-median (pale blue), anterior-lateral (red). **(B)** (Top, Bottom) A s. basicomicum before and after cutting for fluorescent dye introduction, respectively (arrows). **(C)** The representative anterograde staining image of AL. Fluorescent dye was introduced from a single s. basiconicum. **(D)** Distribution of number of glomeruli projected by the basiconic sensillar ORNs among T1-T7 (*n* = 8). In case of **(C)**, all of the 49 stained axons are projected to the T6 region (arrow).

We first constructed glomerular map (Figure 6A), and reconstructed the axonal projection of sensillar ORNs to the AL by the anterograde staining from the lesion of the s. basiconicum (see Figure 6B). The representative staining is shown in Figure 6C. In eight trials of the anterograde staining from the single s. basiconica, the total numbers of stained glomeruli were counted to be 37, 45, 47, 49, 50, 88, 95 and 99, respectively, and regional distribution of those glomeruli in the AL is summarized in Figure 6D. This indicates that the basiconic sensillar ORNs mainly project into the T6 region’s glomeruli.

### Single sensillar recording to the contact stimulation with the repellent

**FIGURE 7:**
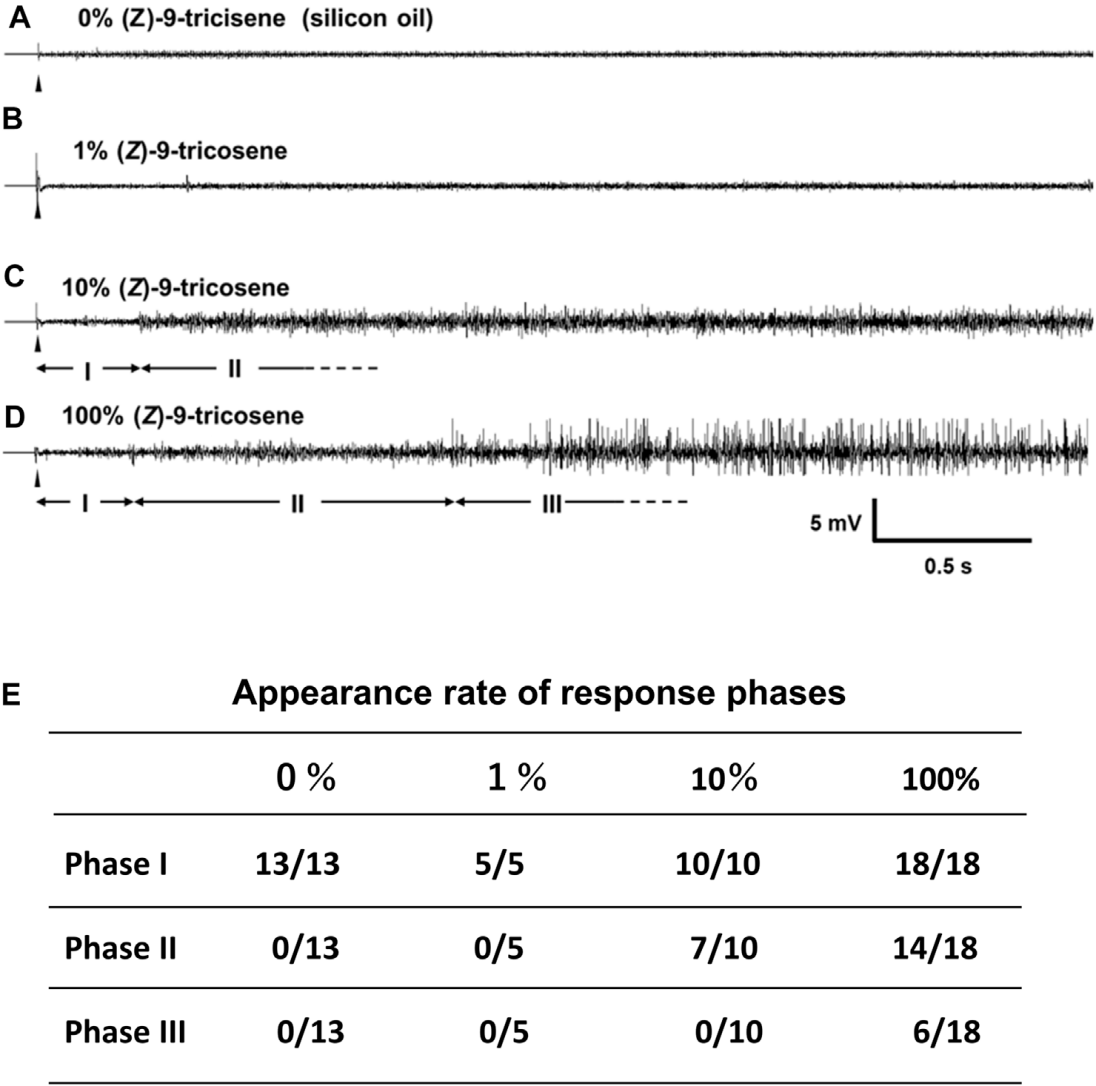
Single sensillar responses of a s. bsiconicum of *C. japonicus* to the contact stimulation with (Z)-9-tricosene. **(A–D)** The representative responses to 0 (silicon oil), 1, 10 and 100% (*Z*)-9-tricosene, respectively. Each trace indicates the first 3-s-AC-recording after beginning of 6-s-stimulation (arrowheads). I, II and III are apparently different phases showing no impulse, multiple impulse units and multiple impulse units including some of large amplitudes, respectively. **(E)** Stimulation-dependent appearance of three phases, I, II and III, presented by the number of recordings including each phase per total number of recordings.

Figure 7 shows a representative electrophysiological response. A series of the single sensillar recording was carried out in a s. basiconicum of *C. japonicus*, which was successively stimulated with 0 (A), 1 (B), 10 (C), or 100% (*Z*)-9-tricosene (D). A and B show the recordings with no impulses (Phase I). C and D show the recordings with relatively small amplitudes of impulses after some latency (Phase II), and D shows further delayed appearance of larger amplitudes of impulses (Phase III). The inset table summarizes, appearance rate of each phase. No impulse was observed in all tested sensilla when stimulated either with 0 (13 sensilla) or 1% (*Z*)-9-tricosene (5 sensilla); while multiple impulse units were recorded after some delay in 7 of 10 and 14 of 18 sensilla stimulated with 10 and 100% (*Z*)-9-tricosene, respectively. Even when the sensilla were stimulated with100% (Z)-9-tricosene, the Phase III were not always appeared but sometimes (6 of 18 sensilla). The timing of phase shift was case by case.

### Barrier effect of the repellent

Besides the laboratory experiments, we conducted 2-weeks’ field experiment using the paired sticky traps with and without barrier of (*Z*)-9-tricosene. Considering the repellent effect of (*Z*)-9-tricosene (see Figure 1), appropriate amount of (*Z*)-9-tricosene should prevent *L. humile* from crossing the barrier. As was expected, the numbers of ants captured on a sticky surface of the trap were significantly reduced, if the trap was surrounded by the barrier of (*Z*)-9-tricosene. We significantly examined statistical comparison in the number of *L. humile* workers captured between the traps in the absence and presence of (*Z*)-9-tricosene barrier, and found significant decrease on day 14 for law dose traps (0.1 mg/cm^2^ (*Z*)-9-tricosene was sprayed) and on day 7 and day14 for high dose traps (1 mg/cm^2^ (*Z*)-9-tricosene was sprayed) (*p* < 0.05, Mann-Whitney’s U test). Other ant species than *L. humile* were hardly trapped in this test field.

### Species selectivity of the repellent

**FIGURE 8:**
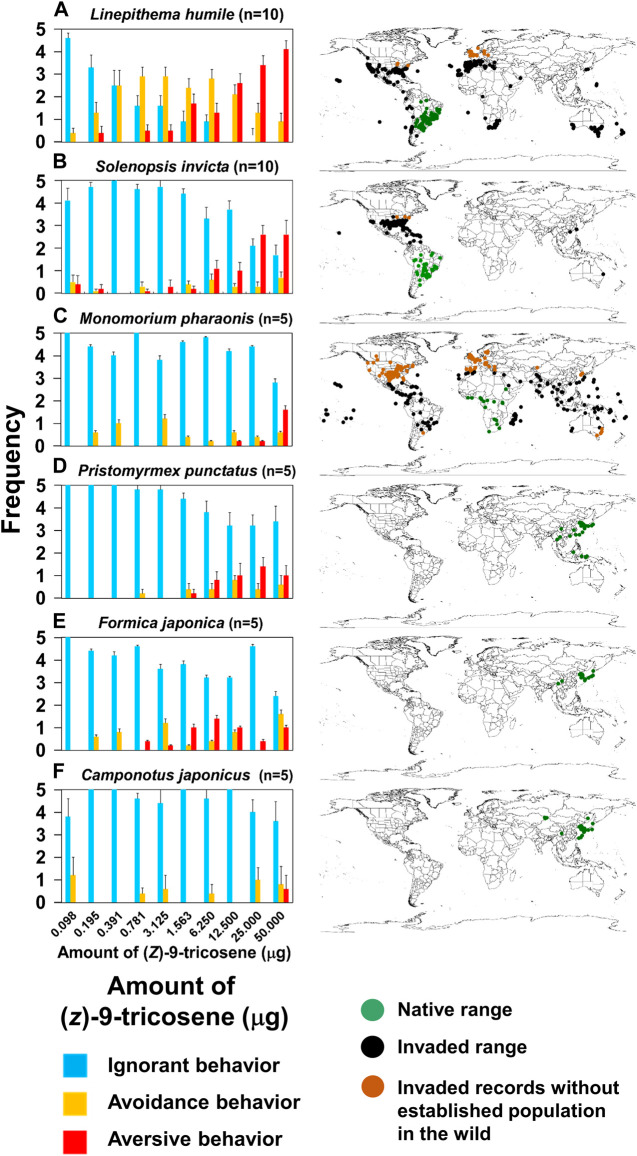
Behavioral effects of (*Z*)-9-tricosene on 6 ant species. **(A–F)** (Left) Date on *L*. *humile*, *S*. *invicta*, *P. punctatus*, *M. pharaonis*, *F. japonica,* and *C. japonicus*. **(A–F)** (Right) Global distributions in 2019. Green and black dots indicate native and invaded ranges, respectively. Brown dots indicate invaded records without established populations (see https://antmaps.org/) (Janicki et al., 2016; Guénard et al., 2017).

To verify the generality of the phenomenon described for *L. humile*, we also measured the behavioral response to (*Z*)-9-tricosene for two other invasive ant species: namely, *Solenopsis invicta* [Myrmicinae] and *Monomorium pharaonis* [Myrmicinae] and three Japanese native species: *Pristomyrmex punctatus* [Myrmicinae], *Formica japonica* [Formicinae] and *C. japonicus* [Formicinae]. The results revealed that (*Z*)-9- tricosene induced aversive behavior in *S. invicta*, similar to that found in *L. humile*, while the response of *M. pharaonis* and the three native species was less frequent and only at high compound doses (Figure 8 Left). Thus, (*Z*)-9-tricosene could also serve as a safe and strong repellent against *S. invicta*, which is another serious invasive species causing damage to agriculture and ecosystems in the invaded areas (see Figure 8 Right).

## Discussion

At the present time, we cannot fully explain why *L. humile* and *S. invicta* demonstrated high sensitivity to (*Z*)-9-tricosene while all the other tested species were not so sensitive as them. The histochemistry-revealed effects of high-dose (*Z*)-9-tricosene in *L. humile* (Figure 3) suggests that it causes an unusual disarray in the perception of odor information in the brain. We found the similar histochemical effect but at 20 times higher doses of (*Z*)-9-tricosene (1mg (*Z*)-9-tricosene on a glass bead) in *C. japonicus* (Figure 4), and we can assume a species-specific difference in physiological resistance to the stimulation with (*Z*)-9-tricosene. By analogy, it is tempting to postulate that other ant species may nonetheless be sensitive to other CHC compound(s).

Given the general understanding that one ORN expresses one olfactory receptor molecule, the question that arises is why does antennal stimulation with a single CHC component activate such a large number of glomeruli in the AL (12–15 glomeruli stained can be seen in Figure 3C) differently from the positive control using benzaldehyde (Figure 5): an unlikely event, even if (*Z*)-9-tricosene can bind to several distinct receptors. Previously, Takeichi et al. (2018) discovered in *C. japonicus* a novel interneuronal network via innexin-mediated electric synapsis that can interconnect 100 or more ORNs within a s. basiconicum in their dendritic processes (Takeichi et al., 2018). According to their mathematic model, the existence of such a network might provide an explanation for our above findings. Consequently, it is predicted that the strong peripheral stimulation even of a single ORN can result in expanded outputs, derived from multiple ORNs projecting to a corresponding multiple number of glomeruli, albeit within a limited region of the ALs possessing convergent axonal projection from the basicoonic sensillar ORNs. The anti-pERK-antibody-staining region of AL in *L. humile* was at the posterior-median position (Figure 3C), which may correspond to the T6 region in *C. japonicus* (Figure 4E). Because of unknown reasons, quantitative evaluation of brightness difference (Figure 4H) to estimate the dose-dependent activation of T6 (Figures 4B−4D) was not very clear, for there is no significant difference in the brightness not only between the contact stimulation conditions with 0 (*n* = 10, Examined glomeruli were in 10 ALs of 5 brains) and 50 mg/bead (*Z*)-9-tricosene (*n* = 12, Examined glomeruli were in 12 ALs of 6 brains) (*p* = 0.314, Mann-Whitney’s U test) but also between that with 0 and 1000 μg/bead (*Z*)-9-tricosene (*n* = 12, Examined glomeruli were in 12 ALs of 6 brains) (*p* = 0.093, Mann-Whitney’s U test).

Considering the interneuronal network hypothesis as a putative explanation for the observed multi-glomerular activation by a single CHC component, (*Z*)-9-tricosene, we tested the larger *C. japonicus*. Although *C. japonicus* is less sensitive to (*Z*)-9-tricosene than *L. humile*, the use of very strong (*Z*)-9-tricosene stimulation had elicited aversive behavior even in *C. japonicus* (Figure 4A; Supplementary Movie S6). In our electrophysiological experiment by a modified “tip-recording” procedure, when the capillary was capped over the sensillum, the lipophilic compound could penetrate into the sensillum via the olfactory pores, and be transported by the chemosensory proteins (carrier for the CHCs) to the receptive membranes of ORNs (Ozaki et al., 2005; Hojo et al., 2015). Consequently, the hundred or more ORNs packed in the sensillum were presumably exposed to the (*Z*)-9-tricosene. When multiple ORNs are directly or indirectly activated via the innexin-involving electric synapse-mediated interneuronal network (Takeichi et al., 2018), the resulting self-generated multiple impulse units can be recorded as shown in Figure 7. When the worker ants were encountered toward large amount of (*Z*)-9-tricosene, in all the stimulated s. basiconica, inward current occurs at every dendritic process of (*Z*)-9-tricosene-sensitive ORNs. Thus, the inward current being divided into neighboring ORNs via electric synapses triggers impulse discharges of multiple units in both directly and indirectly activated ORNs in each single sensillum. The delay in response time (i.e., 0.3 s in Figure 7D), possibly corresponds to the time needed for the transport of the lipophilic stimulus molecules by CSP across the sensillar lymph to reach the receptive ORN membranes in the dendritic processes (Ozaki et al., 2005; Hojo et al., 2015; Takeichi et al., 2018). In view of the results obtained above with *C. japonicus*, we suggest that a similar induction of vigorous response of the phase III will take place upon exposure to (*Z*)- 9-tricosene in the invasive *L. humile* or *S. invicta*, since they are even more sensitive to this compound than *C. japonicus* (Figures 8A,B,F). Figure 6D indicates that the basiconic sensillar ORNs’ axons mainly project to the T6 glomeruli but other glomeruli as well, probably because of technical artifact. It was difficult to cut a single s. basiconicum for the anterograde staining (see Figure 6B). Nonetheless, it is still reliable that the uncharacteristic simultaneous impulse-generating excitation of such a large number of ORNs directly innervating in T6 as shown in Figure 6C, in the absence of additional stimuli such as an apparent threat by encountering ants, may cause an “olfactory hallucination” of the presence of dominant foes, thus eliciting a “touch-and-escape” reaction.

**FIGURE 9:**
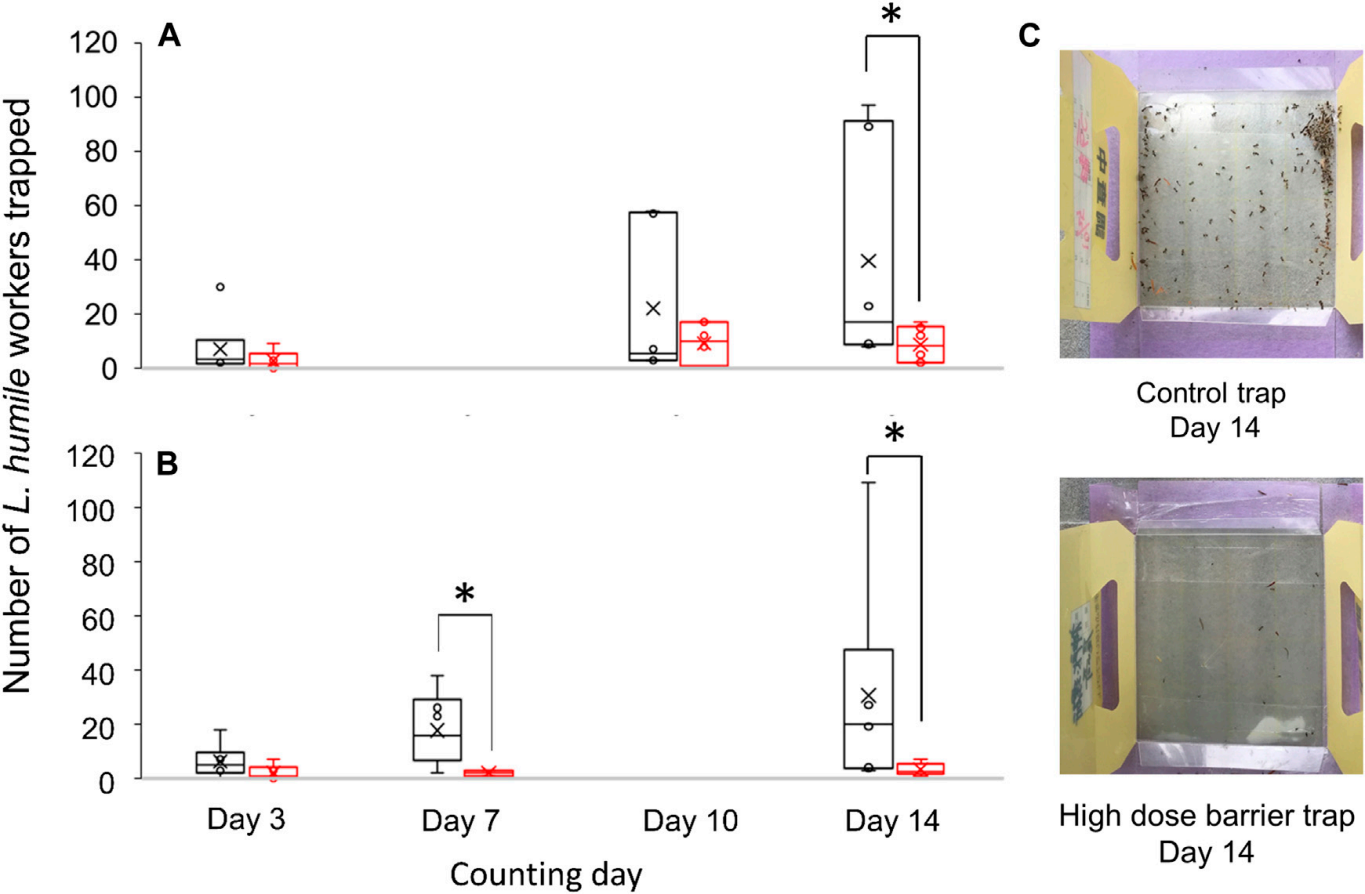
Comparison in the number of *L. humile* workers captured between the traps with or without barrier of (*Z*)-9-tricosene. Field observation was done for 14 days. **(A)** Number of ants captured by the low dose barrier of (*Z*)-9-tricosene trap compared with that by the (*Z*)-9-tricosene-free trap. **(B)** Number of ants captured by the high dose barrier of (*Z*)-9-tricosene trap compared with that by the (*Z*)-9-tricosene-free trap. Asterisks indicate significant difference (*n* = 6, *p* < 0.05, Mann-Whitney’s U test). **(C)** (Top, Bottom) Pictures of the control trap and high dose barrier trap on day 14, respectively.

Recently, we confirmed that the self-glooming of antennae is essential for proper discrimination between nestmates and non-nestmates (Mizutani et al., 2021). We further speculated that, thereafter, the ants start extensive self-grooming of their antennae in order to remove the “olfactory hallucination” that mimics the odor of dominant foes. Two examples of “panic response” to massive amounts of alarm pheromones in interspecific interactions are the propaganda substances used, for example, by slave-making ants (Regnier and Wilson, 1971; Duffield et al., 1976), and the robber stingless bee *Lestrimellita limao* (Blum, 1966). Panic response may also explain why *C. japonicus* responded aversively to its own naturally-occurring (*Z*)-9-tricosene. Ordinarily, this compound is emitted in very small amounts, hence such a minor component could not be used by invasive ants as a chemical sign of existence of native species or chemical cue to avoid the native species. Nevertheless, the aversive response was elicited by exposure to massive amounts. A similar case of panic response (in the form of escape behavior) to its own pheromone was described in *Hypoponera opaciar* (Nick and d'Ettorre, 2012). Actually, (*Z*)-9-tricosene, when applied with adequate quantity, functioned as an effective barrier against *L. humile*, as was suggested by our field experiment (Figure 9). Therefore, it is expected to modify the behavior of *L. humile* and *S. invicta* to keep them away from a treated area and to make a long-lasting (at least for 2 weeks) rigid borderline to separate between invaded and uninvaded or once exterminated areas. We think that (*Z*)-9-tricosene, a less-volatile CHC, would not move those pests far from a treated area, but if they could learn unpleasant place, where they had repeatedly experienced the contact stimulation with (*Z*)-9-tricosene, they might move away.

The aversive effect of (*Z*)-9-tricosene may represent a special case since it was effective against the invasive *L. humile* and *S. invicta* but not against another invasive species, *M. pharaonis* nor against the native ant species studied here. Notwithstanding, we suggest that the above-mentioned species, or indeed any other ant species, may be highly responsive to other CHC components, provided that they possess ORN(s) that are sensitive to such components. There might be species-specific differences in physiological resistance to the unpleasant odor caused by large doses of (*Z*)-9-tricosene, or alike CHC compounds. These may be related to the physiological and/or molecular bases for the characteristic function of the CHC-sensillum or related neural mechanism in the brain, which participate in nestmate/non-nestmate discrimination (Nakanishi et al., 2010; Brandstaetter et al., 2011; Ozaki and Hefetz, 2014; Sharma et al., 2015). We can conclude that ants in general may react aversively to an alien CHC component at appropriate dosages, the specifics of which may depend on the species in question. Through the use of an appropriate aversive compound against an invasive species that is sensitive to it, its population could be contained or even repelled from the invasion area, enabling the less sensitive native species to reoccupy that area. Thus, the species-specific effects of an aversive compound on *L. humile* or *S. invicta* would be beneficial to native species, facilitating the recovery of the previous natural ecosystem and biodiversity.

## Data Availability

The raw data supporting the conclusions of this article will be made available by the authors, without undue reservation.

## Supplementary material

SUPPLEMENTARY MOVIE S1 Cooperative offence by *Linepithema humile* workers against a *Camponotus japonicus* worker.

SUPPLEMENTARY MOVIE S2 Aversive behavior of *Linepithema humile* toward a glass bead (2 mm diameter) coated with six-ant-equivalent of *Camponotus japonicus* cuticular hydrocarbons.

SUPPLEMENTARY MOVIE S3 Ignorant behavior of *Linepithema humile* toward a glass bead (2 mm diameter) treated with 20 μL hexane.

SUPPLEMENTARY MOVIE S4 Avoidance behavior of *Linepithema humile* toward a glass bead (2 mm diameter) coated with 6.25 μg of (*Z*)-9-tricosene.

SUPPLEMENTARY MOVIE S5 Aversive behavior of *Linepithema humile* toward a glass bead (2 mm diameter) coated with 50 μg of (*Z*)-9-tricosene.

SUPPLEMENTARY MOVIE S6 Aversive behavior of *Camponotus japonicus* toward a glass bead (4 mm diameter) coated with 2 mg of (*Z*)-9-tricosene.
