# Supplementary material for: Chemical identification of an active component and putative neural mechanism for repellent effect of a native ant’s odor on invasive species
Source: Front Physiol. 2022 Aug 30;13:844084. doi: 10.3389/fphys.2022.844084 (PMC9468892; doi:10.3389/fphys.2022.844084)
Supplement: Supplementary file 2 [file Table1.DOCX]

**Table of contents**

Supplementary Text···············································································2

1. Synthesis of unsaturated hydrocarbons····················································2

2-1. Synthesis of (R)-5-methylheptacosane·················································9

2-2. Synthesis of (R)-13-methylheptacosane···············································12

3. Synthesis of dimethyl-branched alkane, (7R,15S)-7,15-dimethylheptacosane·····15

4. Synthesis of trimethyl-branched alkanes·················································22

References························································································43

**Supplementary Text**

**1. Synthesis of unsaturated hydrocarbons**

The unsaturated hydrocarbons were chemically synthesized except commercially available (*Z*)-9-tricosene. The *Z* double bonds were introduced by hydrogenation of the alkyne over Lindlar’s catalyst. The precursor internal alkynes were synthesized as follows. The terminal alkynes were deprotonated by butyl lithium, and the alkynyl lithiums thus formed were reacted with the alkyl iodides. Butyl lithium was the limiting reagent in this reaction. Excess terminal alkynes and the alkyl iodides were removed by vigorous evaporation and treatment with silica gel impregnated with silver nitrate, respectively. In the said process alkyl iodides were turned to the corresponding nitro compounds, which could be readily separated by chromatography on silica gel. The pure alkynes thus obtained were hydrogenated over Lindlar’s catalyst. The reactions were carefully monitored by GC analysis and the complete consumption of the alkynes was confirmed. The mixture of the alkenes and the over-reduced product alkanes were separated by chromatography on silica gel impregnated with silver nitrate.

Preparation of silica gel impregnated with silver nitrate (*36*)

A solution of silver nitrate (22 g) in water (120 mL) was poured to silica gel (200 g, 40–63 μm, pore size 6 nm). The suspension was vigorously stirred for 5 min and dried in an oven at 200 ^o^C for 18 h, resulting in almost white powder.

General procedure to synthesize the alkyl iodides from the alkyl bromides (*37*)

To a solution of alkyl bromide (13.7 mmol) in acetone (93 mL) was added NaI (110 mmol). The reaction was refluxed for 2 h and cooled to room temperature. The mixture was extracted with diethyl ether and washed with water, Na_2_S_2_O_3_ aq. and brine. The organic layer was dried over MgSO_4_. After filtration, the filtrate was dried in vacuo to afford the alkyl iodides, which were analytically pure and used without further purification.

General procedure to synthesize the alkyl iodides from the alcohols (*38*)

To a mixture of CH_3_CN (66 mL) and diethyl ether (66 mL) was added alcohol (9.6 mmol), imidazole (13.4 mmol), triphenylphosphine (12.5 mmol) and iodine (13.4 mmol) in this order. The reaction was stirred at 20 ^o^C for 16 h, before Na_2_S_2_O_3_ aq. was added. The layers were partitioned and the aqueous layer was extracted with diethyl ether three times. The combined organic layer was dried over MgSO_4_, dried in vauo and purified by chromatography on silica gel (hexane as an eluent).

General procedure to synthesize the internal aliphatic alkynes (*39*)

To a solution of terminal alkyne (3.6 mmol) in tetrahydrofuran (16 mL) was added n-BuLi (1.6 M in hexane, 3.4 mmol) at 0 ^o^C. The mixture was stirred for 20 min at 0 ^o^C and allowed to warm to 20 ^o^C, before alkyl iodide (5.1 mmol) was added. The reaction mixture was refluxed for 15 h. The reaction was quenched by the addition of saturated NH_4_Cl aq. The mixture was extracted with diethyl ether three times, washed with NH_4_Cl aq., water and brine and dried over MgSO_4_. After filtration, the filtrate was dried in vacuo until the remaining terminal alkyne was evaporated. The residue was dissolved in hexane (10 mL), to which silica gel impregnated with silver nitrate (5.0 g) was added. The mixture was stirred at 20 ^o^C for 2 h. After filtration, the filtrate was dried in vacuo and purified by chromatography on silica gel (hexane as an eluent) to afford the internal aliphatic alkyne.

General procedure to synthesize the (*Z*)-alkenes from the internal aliphatic alkynes (*40*)

To a solution of quinoline (0.16 mmol, 18 mol%) and the internal alkyne (0.86 mmol) in MeOH (3.5 mL) was added 5% Pd/CaCO_3_ poisoned with lead (from TCI, 2.6 μmol, 11 mg). Hydrogen gas was introduced and the hydrogen atmosphere was maintained with a balloon filled with hydrogen gas. The reaction was stirred at 50 ^o^C until all the alkyne was consumed (typically 2–4 h). After the filtration over celite, the filtrate was concentrated and purified by chromatography on silica gel impregnated with silver nitrate (hexane as an eluent).

7-tricosyne

MS (m/z, abundance): 320 (2, M^+^), 166(3), 137 (4), 123 (10), 109 (32), 95 (70), 81 (100), 67 (94), 55 (47).

(*Z*)-7-tricosene

MS (m/z, abundance): 322 (12, M+), 167 (2), 153 (4), 139 (6), 125 (17), 111 (39), 97 (84), 83 (100), 69 (94).

9-hexacosyne

MS (m/z, abundance): 194 (2, M-168), 151 (3), 137 (10), 123 (14), 109 (24), 95 (65), 81 (100), 67 (69), 55 (45).

(*Z*)-9-hexacosene

MS (m/z, abundance): 207 (4, M-157), 153 (2), 139 (6), 125 (10), 111 (23), 97 (53), 83 (62), 69 (72), 55 (100).

9-tricosyne

MS (m/z, abundance): 207 (2, M-113), 194 (2), 151 (3), 137 (8), 123 (14), 109 (22), 95 (62), 81 (100), 67 (85), 55 (60).

7-hexacosyne

MS (m/z, abundance): 362 (2, M^+^), 264 (2), 166 (4), 137 (4), 123 (12), 109 (34), 95 (72), 81 (100), 67 (83), 55 (40).

(*Z*)-7-hexacosene

MS (m/z, abundance): 181 (2, M-179), 167 (3), 153 (6), 139 (8), 125 (20), 111 (47), 97 (96), 83 (100), 69 (91), 57 (92).

9-pentacosyne

MS (m/z, abundance): 235 (3, M-113), 194 (7), 166 (6), 151 (8), 137 (22), 123 (26), 109 (34), 95 (78), 81 (100), 67 (69), 55 (43).

(*Z*)-9-pentacosene

MS (m/z, abundance): 181 (2, M-169), 167 (4), 153 (6), 139 (12), 125 (24), 111 (54), 97 (100), 83 (96), 69 (75), 57 (85).

9-heptacosyne

MS (m/z, abundance): 263 (2, M-113), 207 (7), 194 (6), 166 (4), 151 (5), 137 (24), 124 (24), 109 (39), 95 (84), 81 (100), 67 (63), 55 (40).

(*Z*)-9-heptacosene

MS (m/z, abundance): 378 (3, M^+^), 206 (6), 181 (6), 167 (7), 153 (10), 139 (16), 125 (36), 111 (66), 97 (100), 83 (87), 69 (71), 57 (78).

7-pentacosyne

MS (m/z, abundance): 166 (4, M-182), 137 (6), 123 (14), 109 (38), 95 (70), 81 (100), 67 (80), 55 (40).

(*Z*)-7-pentacosene

MS (m/z, abundance): 350 (4, M^+^), 167 (3), 153 (5), 139 (7), 125 (17), 111 (42), 97 (84), 83 (100), 57 (98).

7-heptacosyne

MS (m/z, abundance): 291 (2, M-85), 207 (7), 180 (4), 166 (12), 151 (6), 137 (10), 123 (26), 109 (49), 95 (94), 81 (100), 67 (70), 55 (40).

(*Z*)-7-heptacosene

MS (m/z, abundance): 378 (2, M^+^), 207 (8), 181 (5), 167 (6), 153 (8), 139 (16), 125 (34), 111 (62), 97 (100), 83 (84), 69 (72), 57 (76).

9-nonacosyne

MS (m/z, abundance): 208 (2, M-196), 194 (8), 166 (4), 151 (8), 137 (24), 109 (38), 95 (84), 81 (100), 67 (66), 55 (42).

(*Z*)-9-nonacosene

MS (m/z, abundance): 406 (3, M^+^), 209 (4), 195 (6), 181 (8), 167 (10), 153 (12), 139 (18), 125 (35), 111 (54), 97 (100), 83 (86), 69 (69), 57 (81).

7-nonacosyne

MS (m/z, abundance): 207 (2, M-197), 180 (4), 166 (10), 151 (6), 137 (10), 123 (24), 109 (60), 95 (94), 81 (100), 67 (70), 55 (39).

(*Z*)-7-nonacosene

MS (m/z, abundance): 406 (1, M^+^), 195 (2), 181 (4), 167 (6), 153 (10), 139 (12), 125 (28), 111 (52), 97 (100), 83 (86), 69 (70), 57 (76).

**2. Synthesis of monomethyl-branched alkanes**

Both enantiomers of 13-methylheptacosane and 5-methylheptacosane were synthesized. The synthesis of *R* enantiomers of the compounds is shown. The *S* enantiomers were synthesized in the same manner.

**2-1. Synthesis of (*R*)-5-methylheptacosane**

(3*R*)-3,7-dimethyloct-6-en-1-yl 4-methylbenzene-1-sulfonate

Synthesized according to the literature. (*41*)

(4*S*)-4-methylhexacosanal

Magnesium turnings (793 mg, 32.6 mmol) was placed in a round-bottomed flask and dried using a heat-gun under vacuum. After argon was introduced, THF (4 mL) and 1,2-dibromoethane (84 μL, 3 mol% to Mg) were added. After the gas evolution was ceased, the flask was put into the 90 °C oil bath. To the refluxed mixture was then added 1-bromoicosane (8.73 g, 24.2 mmol) in THF (23 mL) over 10 min. The mixture was kept refluxed for 2 hours. The hot supernatant was transferred to another flask via a cannula. THF (12 mL) was added and the mixture was cooled to −78 °C (the reaction mixture was frozen at this temperature). To the reaction mixture were added a solution of (3*R*)-3,7-dimethyloct-6-en-1-yl 4-methylbenzene-1-sulfonate (2.5 g, 8.1 mmol) in THF (5 mL) and Li_2_CuCl_4_ in THF (0.1 M, 0.8 mL, 1 mol% to the tosylate). The frozen mixture was warmed to 0 °C and stirred for 20 h. The mixture was poured into sat. NH_4_Cl aq. (150 mL) and extracted thrice with hexane. The combined organic layer was dried over MgSO_4_, filtered and concentrated in vacuo. The residue was purified by chromatography on SiO_2_ (hexane as an eluent) to give a mixture of desired intermediate (6*S*)-2,6-dimethyloctacos-2-ene and alkanes that are mainly derived from the unreacted organomagnesium reagent.

To the mixture obtained above were added hexane (80 mL), CH_2_Cl_2_ (130 mL), MeOH (15 mL), and NaHCO_3_ (677 mg). The mixture was cooled to −78 °C and a mixed gas of O_2_ and O_3_ was bubbled until the blue color persisted (ca. 2.5 h later). Then argon was bubbled at −78 °C until the blue color disappeared. Dimethylsulfide (8.9 mL) was added at −78 °C and the mixture was warmed to rt and kept stirred for 16 h. The mixture was poured into water (100 mL) and extracted twice with CH_2_Cl_2_ and once with hexane. The combined organic layer was dried over MgSO_4_, filtered and concentrated in vacuo. The residue was purified by chromatography on SiO_2_ to give (4*S*)-4-methylhexacosanal (2.09 g, 66% yield).

[α]^28^_D_ = +0.95 (c 1.27, CHCl_3_)

^1^H NMR (400 MHz,CDCl_3_): δ = 9.77 (t, *J* = 1.9 Hz, 1H), 2.51–2.33 (m, 2H), 1.78–1.57 (m, 1H), 1.51–1.37 (m, 2H), 1.37–1.05 (m, 42H), 0.92–0.83 (m, 6H).

^13^C NMR (100MHz, CDCl_3_); δ = 203.0, 41.7, 36.7, 32.4, 31.9, 29.9, 29.7, 29.4, 28.9, 27.0, 22.7, 19.4, 14.1.

(5*S*)-5-methylheptacos-1-ene

To a solution of methyl(triphenyl)phosphanium bromide (590 mg, 1.65 mmol) in THF (20 mL) was added at −78 °C nBuLi (1.6 M in hexane, 0.95 mL, 1.52 mmol). The mixture was warmed to rt and stirred at rt for 30 min, then cooled to −78 °C. To the mixture was added a solution of (4*S*)-4-methylhexacosanal (500 mg, 1.27 mmol) in THF (6 mL). The mixture was warmed to rt and stirred for 3 h. The reaction was quenched by addition of sat. NH_4_Cl aq. and the mixture was extracted with Et_2_O. The organic layer was dried over MgSO_4_, filtered and concentrated in vacuo. The residue was purified by chromatography on SiO_2_ to give (5*S*)-5-methylheptacos-1-ene (280 mg, 56% yield).

[α]^28^_D_ = −0.44 (c 1.08, CHCl_3_)

^1^H NMR (400 MHz,CDCl_3_): δ = 5.81 (ddt, *J* = 17.0, 10.3, 6.6 Hz, 1H), 4.99 (dq, *J* = 17.0, 1.6 Hz, 1H), 4.92 (ddt, 10.3, 2.1, 1.0 Hz, 1H), 2.17–1.94 (m, 2H), 1.48–1.03 (m, 45H), 0.95–0.81 (m, 6H).

^13^C NMR (100MHz, CDCl_3_); δ = 139.5, 113.9, 37.0, 36.3, 32.3, 32.0, 31.4, 30.0, 29.7, 29.7, 29.4, 27.0, 22.7, 19.5, 14.1.

(5*R*)-5-methylheptacosane

To a solution of (5*S*)-5-methylheptacos-1-ene (200 mg, 0.51 mmol) in hexane (14 mL) was added 10% Pd/C (wet, 25 mg). The atmosphere was replaced to H_2_ and kept by using a balloon. The reaction mixture was stirred at rt for 2.5 h, then filtered over celite and concentrated in vacuo. The residue was further purified by removing a trace amount of volatile compounds with a Kugelrohr apparatus, affording (5*R*)-5-methylheptacosane (147.3 mg, 73% yield).

[α]^28^_D_ = +0.75 (c 1.17, CHCl_3_)

^1^H NMR (400 MHz,CDCl_3_): δ = 1.47–0.99 (m, 49H), 0.89 (t, *J* = 6.8 Hz, 3H), 0.88 (t, *J* = 7.0 Hz, 3H), 0.84 (d, *J* = 6.5 Hz, 3H).

^13^C NMR (100MHz, CDCl_3_); δ = 37.1, 36.8, 32.8, 32.0, 30.1, 29.7, 29.7, 29.4, 29.4, 27.1, 23.1, 22.7, 19.7, 14.2, 14.1.

MS (m/z, abundance): 337 (14, M-57), 308 (3), 267 (2), 253 (3), 207 (4), 169 (4), 141 (6), 127 (9), 113 (12), 99 (18), 85 (100), 57 (94).

**2-2. Synthesis of (*R*)-13-methylheptacosane**

(4*S*)-4-methyloctadecanal

Magnesium turnings (793 mg, 32.6 mmol) was placed in a round-bottomed flask and dried using a heat-gun under vacuum. After argon was introduced, THF (4 mL) and 1,2-dibromoethane (84 μL, 3 mol% to Mg) were added. After the gas evolution was ceased, the flask was put into the 90 °C oil bath. To the refluxed mixture was then added 1-bromododecane (5.8 mL, 24.2 mmol) in THF (23 mL) over 25 min. The mixture was kept refluxed for 2 hours. The hot supernatant was transferred to another flask via a cannula. THF (12 mL) was added and the mixture was cooled to −78 °C (the reaction mixture was frozen at this temperature). To the reaction mixture were added a solution of (3*R*)-3,7-dimethyloct-6-en-1-yl 4-methylbenzene-1-sulfonate (2.5 g, 8.1 mmol) in THF (5 mL) and Li_2_CuCl_4_ in THF (0.1 M, 0.8 mL, 1 mol% to the tosylate). The frozen mixture was warmed to 0 °C and stirred for 42 h. The mixture was poured into sat. NH_4_Cl aq. (150 mL) and extracted thrice with hexane. The combined organic layer was dried over MgSO_4_, filtered and concentrated in vacuo. The residue was purified by chromatography on SiO_2_ (hexane as an eluent) to give a mixture of desired intermediate (6*S*)-2,6-dimethylicos-2-ene and alkanes that are mainly derived from the unreacted organomagnesium reagent.

To the mixture obtained above were added hexane (80 mL), CH_2_Cl_2_ (130 mL), MeOH (15 mL), and NaHCO_3_ (677 mg). The mixture was cooled to −78 °C and a mixed gas of O_2_ and O_3_ was bubbled until the blue color persisted (ca. 2.5 h later). Then argon was bubbled at −78 °C until the blue color disappeared. Dimethylsulfide (8.9 mL) was added at −78 °C and the mixture was warmed to rt and kept stirred for 34 h. The mixture was poured into water (100 mL) and extracted twice with CH_2_Cl_2_ and once with hexane. The combined organic layer was dried over MgSO_4_, filtered and concentrated in vacuo. The residue was purified by chromatography on SiO_2_ to give (4*S*)-4-methyloctadecanal (2.05 g, 90% yield).

[α]^28^_D_ = −1.11 (c 1.23, CHCl_3_)

^1^H NMR (400 MHz,CDCl_3_): δ = 9.77 (t, *J* = 1.88 Hz, 1H), 2.52–2.30 (m, 2H), 1.77–1.59 (m, 1H), 1.49–1.37 (m, 2H), 1.36–1.04 (m, 26H), 0.94–0.83 (m, 6H).

^13^C NMR (100MHz, CDCl_3_); δ = 203.0, 41.7, 36.7, 32.4, 31.9, 29.9, 29.7, 29.7, 29.4, 28.9, 27.0, 22.7, 19.4, 14.1.

(13*S*)-13-methylheptacos-9-ene

To a solution of nonyl(triphenyl)phosphanium bromide (864.1 mg, 1.85 mmol) in THF (24 mL) was added at −78 °C nBuLi (1.6 M in hexane, 1.07 mL, 1.70 mmol). The mixture was warmed to rt and stirred at rt for 30 min, then cooled to −78 °C. To the mixture was added a solution of (4*S*)-4-methyloctadecanal (400 mg, 1.42 mmol) in THF (5 mL). The mixture was warmed to rt and stirred for 5.5 h. The reaction was quenched by addition of sat. NH_4_Cl aq. and the mixture was extracted with Et_2_O. The organic layer was dried over MgSO_4_, filtered and concentrated in vacuo. The residue was purified by chromatography on SiO_2_ to give (13*S*)-13-methylheptacos-9-ene (*E-Z* mixture, 470 mg, 84% yield).

(13*R*)-13-methylheptacosane

To a solution of (13*S*)-13-methylheptacos-9-ene (200 mg, 0.51 mmol) in hexane (14 mL) was added 10% Pd/C (wet, 25 mg). The atmosphere was replaced to H_2_ and kept by using a balloon. The reaction mixture was stirred at rt for 4.5 h, then filtered over celite and concentrated in vacuo. The residue was further purified by removing a trace amount of volatile compounds with a Kugelrohr apparatus, affording (13*R*)-13-methylheptacosane (173.9 mg, 87% yield).

[α]^27^_D_ = +0.12 (c 0.89, CHCl_3_)

^1^H NMR (400 MHz,CDCl_3_): δ = 1.45–1.15 (m, 47H), 1.15–1.00 (m, 2H), 0.88 (t, *J* = 6.6 Hz, 6H), 0.83 (d, *J* = 6.5 Hz, 3H).

^13^C NMR (100MHz, CDCl_3_); δ = 37.1, 32.8, 32.0, 30.1, 29.8, 29.7, 29.7, 29.4, 27.1, 22.7, 19.7, 14.1.

MS (m/z, abundance): 379 (2, M-15), 224 (10), 196 (17), 141 (6), 127 (10), 113 (12), 99 (20), 85 (54), 71(76), 57(100).

**3. Synthesis of dimethyl-branched alkane, (7*R*,15*S*)-7,15-dimethylheptacosane**

(4*S*)-4-methyl-6-[(oxan-2-yl)oxy]hexanal

Synthesized according to the literature. (*42*)

(3*S*)-3-methylpentadec-6-en-1-ol

To a solution of nonyl(triphenyl)phosphanium bromide (9.0 g, 19.3 mmol) in THF (150 mL) was added at −78 °C nBuLi (1.6 M in hexane, 11.2 mL, 17.8 mmol). The mixture was warmed to rt and stirred at rt for 30 min, then cooled to −78 °C. To the mixture was added a solution of (4*S*)-4-methyl-6-[(oxan-2-yl)oxy]hexanal (3.2 g, 14.8 mmol) in THF (5 mL). The mixture was warmed to rt and stirred for 11 h. The reaction was quenched by addition of sat. NH_4_Cl aq. and the mixture was extracted with Et_2_O. The organic layer was dried over Na_2_SO_4_, filtered and concentrated in vacuo. The residue was briefly purified by chromatography on SiO_2_ to remove most of triphenylphosphine oxide.

The obtained crude material, 2-{[(3S)-3-methylpentadec-6-en-1-yl]oxy}oxane, was dissolved in THF (100 mL). To the solution was added at rt 1M HCl aq. (10 mL, 10 mmol). The mixture was stirred at rt for 10 h. The reaction was quenched by addition of sat. NaHCO_3_ aq., and then concentrated in vacuo to remove THF. The residue was dissolved in CH_2_Cl_2_ and washed with water. The water layer was extracted with CH_2_Cl_2_ twice, then the combined organic layer was dried over Na_2_SO_4_, filtered and concentrated in vacuo. The residue was purified by chromatography on SiO_2_ to give (3*S*)-3-methylpentadec-6-en-1-ol (1.7 g, 63% yield in two steps) as a *E-Z* mixture.

^1^H NMR (400 MHz,CDCl_3_): δ = 5.42–5.28 (m, 2H), 3.76–3.62 (m, 2H), 2.14–1.92 (m, 4H), 1.68–1.53 (m, 2H), 1.46–1.16 (m, 17H), 0.94–0.86 (m, 6H).

(3*S*)-3-methylpentadecan-1-ol

To a solution of (3*S*)-3-methylpentadec-6-en-1-ol (1.65 g, 6.86 mmol) in hexane (60 mL) was added 10% Pd/C (wet, 210 mg). The atmosphere was replaced to H_2_ and kept by using a balloon. The reaction mixture was stirred at rt for 3 h, then filtered over celite and concentrated in vacuo, affording (3*S*)-3-methylpentadecan-1-ol (1.4 g, 82% yield).

[α]^27^_D_ = –0.93 (c 0.98, CHCl_3_)

^1^H NMR (400 MHz,CDCl_3_): δ = 3.74–3.62 (m, 2H), 1.66–1.10 (m, 25H), 0.91–0.83 (m, 6H).

^13^C NMR (100MHz, CDCl_3_); δ = 61.3, 40.0, 37.1, 31.9, 30.0, 29.7, 29.7, 29.6, 29.5, 29.4, 27.0, 22.7, 19.7, 14.1.

(3*S*)-3-methylpentadecyl 4-methylbenzene-1-sulfonate

To a solution of (3*S*)-3-methylpentadecan-1-ol (1.0g, 4.1 mmol) in pyridine (3.9 mL) was added at 0 °C TsCl (1.0 g, 5.3 mmol). The mixture was stirred at 0 °C for 7 h, then quenched by addition of 4M HCl aq. The mixture was extracted with Et_2_O. The organic layer was washed with 4M HCl aq. twice, NaHCO_3_ aq. once, and dried over Na_2_SO_4_. The mixture was filtered and concentrated in vacuo to give (3*S*)-3-methylpentadecyl 4-methylbenzene-1-sulfonate (1.1 g, 68% yield).

[α]^27^_D_ = –0.42 (c 1.0, CHCl_3_)

^1^H NMR (400 MHz,CDCl_3_): δ = 7.79 (d, *J =* 8.4 Hz, 2H), 7.34 (d, *J* = 8.4 Hz, 2H), 4.11–4.01 (m, 2H), 2.45 (s, 3H), 1.71–0.97 (m, 25H), 0.88 (t, *J* = 6.8 Hz, 3H), 0.80 (d, *J* = 6.4 Hz, 3H).

^13^C NMR (100MHz, CDCl_3_); δ = 144.6, 133.3, 129.8, 127.9, 69.1, 36.6, 35.7, 31.9, 29.8, 29.7, 29.7, 29.4, 29.2, 26.8, 22.7, 21.6, 19.2, 14.1.

(3*S*)-1-iodo-3-methylpentadecane

To a solution of (3*S*)-3-methylpentadecyl 4-methylbenzene-1-sulfonate (401.8 mg, 1.0 mmol) in acetone (10 mL) was added NaI (320 mg, 2.1 mmol). The suspension was stirred at 75 °C for 15 h. The cooled reaction mixture was concentrated in vacuo, and the obtained residue was suspended in hexane. The mixture was washed with water, dried over Na_2_SO_4_. The mixture was filtered and concentrated in vacuo to give (3*S*)-1-iodo-3-methylpentadecane (356 mg, 95% yield). The obtained iodide was immediately used for the next reaction.

^1^H NMR (400 MHz,CDCl_3_): δ = 3.29–3.13 (m, 2H), 1.92–1.82 (m, 1H), 1.70–1.58 (m, 1H), 1.57–1.47 (m, 1H), 1.34–1.05 (m, 22H), 0.92–0.82 (m, 6H).

(6*R*)-2,6-dimethyldodec-2-ene (*43*)

To a suspension of CuI (9.8 g, 51.5 mmol) in Et_2_O (72 mL) was added at −30 °C nBuLi (1.6 M in hexane, 64 mL, 103 mmol). (*44*) The reaction mixture was stirred at −30 °C for 1 h, then cooled to −78 °C. At that temperature, (3*S*)-3,7-dimethyloct-6-en-1-yl 4-methylbenzene-1-sulfonate (8.0 g, 25.8 mmol) in Et_2_O (50 mL) was added, and the mixture was stirred at −35 °C for 2 h. The reaction was quenched by addition of sat. NH_4_Cl aq. and filtered over celite. The filtrate was washed with water, dried over Na_2_SO_4_. The mixture was filtered and concentrated in vacuo to give (6*R*)-2,6-dimethyldodec-2-ene (5.3 g, quant).

[α]^27^_D_ = –0.75 (c 0.97, CHCl_3_).

^1^H NMR (400 MHz,CDCl_3_): δ = 5.10 (apparent t, *J* = 7.1 Hz, 1H), 2.06–1.87 (m, 2H), 1.68 (s, 3H), 1.60 (s, 3H), 1.46–1.18 (m, 11H), 1.18–1.07 (m, 2H), 0.91–0.84 (m, 6H).

^13^C NMR (100MHz, CDCl_3_); δ = 130.9, 125.1, 37.1, 37.0, 32.4, 32.0, 29.7, 27.0, 25.7, 25.6, 22.7, 19.6, 17.6, 14.1.

(4*R*)-4-methyldecan-1-ol

To a solution of (6*R*)-2,6-dimethyldodec-2-ene (5.0 g, 25.3 mmol) in CH_2_Cl_2_ (127 mL) were added MeOH (10 mL) and NaHCO_3_ (2.12 g, 25.3 mmol). The mixture was cooled to −78 °C and a mixed gas of O_2_ and O_3_ was bubbled until the blue color persisted (ca. 5 h later). Then argon was bubbled at −78 °C until the blue color disappeared. Dimethylsulfide (5.0 mL) was added at −78 °C and the mixture was warmed to rt and kept stirred for 11.5 h. The mixture was concentrated in vacuo, then Et_2_O (150 mL) was added. The mixture was washed with water thrice. The combined organic layer was dried over Na_2_SO_4_, filtered and concentrated in vacuo to give a crude material of (4*R*)-4-methyldecanal (5.18 g).

A portion of crude (4*R*)-4-methyldecanal (2.50 g) was dissolved in MeOH (50 mL) and cooled to 0 °C. To the solution was added NaBH_4_ (0.56 g, 12.7 mmol). The reaction was stirred for 5 h, then concentrated in vacuo. Then water (50 mL) was added to the obtained residue and extracted thrice with CH_2_Cl_2_. The combined organic layer was dried over Na_2_SO_4_. The mixture was filtered and concentrated in vacuo to give (4*R*)-4-methyldecan-1-ol (1.84 g, corresponding to 84% 2-step yield from (6*R*)-2,6-dimethyldodec-2-ene).

[α]^27^_D_ = +1.3 (c 0.98, CHCl_3_)

^1^H NMR (400 MHz,CDCl_3_): δ = 3.63 (t, *J* = 6.4 Hz, 2H), 1.66–1.48 (m, 2H), 1.48–1.04 (m, 14H), 0.88 (t, *J* = 6.6 Hz, 3H), 0.87 (d, *J* = 6.5 Hz, 3H).

(4*R*)-4-methyldecyl 4-methylbenzene-1-sulfonate

To a solution of (4*R*)-4-methyldecan-1-ol (1.5 g, 8.7 mmol) in pyridine (7.8 mL) was added at 0 °C TsCl (2.16 g, 11.3 mmol). After stirred for 19 h at 0 °C, the reaction was quenched by addition of 4M HCl. The mixture was extracted with Et_2_O, and washed with 4M HCl twice and NaHCO_3_ once. The organic layer was dried over Na_2_SO_4_. The mixture was filtered and concentrated in vacuo to give (4*R*)-4-methyldecyl 4-methylbenzene-1-sulfonate (2.36 g, 83% yield).

[α]^27^_D_ = –2.7 (c 1.0, CHCl_3_)

^1^H NMR (400 MHz,CDCl_3_): δ = 7.79 (d, *J* = 8.3 Hz, 2H), 7.34 (d, *J* = 8.0 Hz, 2H), 4.01 (t, *J* = 6.6 Hz, 2H), 2.45 (s, 3H), 1.73–1.54 (m, 2H), 1.38–1.00 (m, 13H), 0.88 (t, *J* = 7.0 Hz, 3H), 0.80 (d, *J* = 6.5 Hz, 3H).

^13^C NMR (100MHz, CDCl_3_); δ = 144.6, 133.3, 129.8, 127.9, 77.1, 36.8, 32.5, 32.3, 31.9, 29.6, 26.9, 26.5, 22.7, 21.6, 19.6, 19.4, 14.1.

(6*R*)-6-methyldodec-1-yne

To a solution of (4*R*)-4-methyldecyl 4-methylbenzene-1-sulfonate (1.51 g, 4.60 mmol) in DMSO (7.5 mL) was added at −20 °C lithium acetylide ethylenediamine complex (630 mg, 6.89 mmol). After stirred at rt for 6 h, the reaction was quenched by addition of water. The mixture was extracted with hexane, and the organic layer was washed with water twice, and dried over Na_2_SO_4_. The mixture was filtered, concentrated in vacuo and purified by chromatography on SiO_2_ to give (6*R*)-6-methyldodec-1-yne (0.53 g, 65% yield).

^1^H NMR (400 MHz,CDCl_3_): δ = 2.16 (dt, *J* = 2.6, 7.2 Hz, 2H), 1.93 (t, *J* = 2.6 Hz, 1H), 1.64–1.02 (m, 15H), 0.93–0.83 (m, 6H).

^13^C NMR (100MHz, CDCl_3_); δ = 84.8, 68.0, 36.9, 36.2, 32.4, 31.9, 29.7, 27.0, 26.1, 22.7, 19.6, 18.7, 14.1.

(7*R*,15*S*)-7,15-dimethylheptacosane

To a solution of (6*R*)-6-methyldodec-1-yne (202.7 mg, 1.12 mmol) in THF (0.7 mL) was added hexamethylphosphoramide (HMPA, 0.16 mL). To the mixture was added at −70 °C nBuLi (1.6 M in hexane, 0.76 mL, 1.22 mmol), warmed to −10 °C, and then cooled to −70 °C. A solution of (3*S*)-1-iodo-3-methylpentadecane (333.4 mg, 0.95 mmol) in THF (0.6 mL) was added to the reaction. The mixture was warmed to 75 °C over 1 h, and stirred at 75 °C for 13 h. After cooled to rt, the reaction was quenched by addition of water and extracted with hexane. The organic layer was washed with water thrice, dried over Na_2_SO_4_. The mixture was filtered, concentrated in vacuo and purified by chromatography on SiO_2_ to give crude (7*R*,15*S*)-7,15-dimethylheptacos-11-yne (304 mg, mixture with (6*R*)-6-methyldodec-1-yne).

A part of the crude material of (7*R*,15*S*)-7,15-dimethylheptacos-11-yne (91% purity, 200 mg) was dissolved in hexane (5 mL) and 10% Pd/C (wet, 30 mg) was added. The atmosphere was replaced to H_2_ and kept by using a balloon. The reaction mixture was stirred at rt for 2 h, then filtered over celite and concentrated in vacuo. The residue was further purified by removing a trace amount of volatile compounds with a Kugelrohr apparatus, affording (7*R*,15*S*)-7,15-dimethylheptacosane (173.7 mg, corresponding to 68% 2-step yield from (3*S*)-1-iodo-3-methylpentadecane).

[α]^27^_D_ = +0.21 (c 0.96, CHCl_3_)

^1^H NMR (400 MHz,CDCl_3_): δ = 1.43–1.16 (m, 44H), 1.15–1.00 (m, 4H), 0.88 (apparent t, *J* = 6.2 Hz, 6H), 0.83 (d, *J* = 6.5 Hz, 6H).

^13^C NMR (100MHz, CDCl_3_); δ = 37.1, 32.8, 32.0, 31.9, 30.0, 29.8, 29.8, 29.7, 29.7, 29.4, 27.1, 27.1, 22.7, 19.7, 14.1.

MS (m/z, abundance): 395 (M^+^-15, 4), 379 (3), 323 (16), 295 (4), 239 (18), 196 (26), 183 (6), 153 (13), 141 (16), 112 (40), 99 (32), 85 (67), 71 (100), 57 (72).

**4. Synthesis of trimethyl-branched alkanes**

(2*S*,4*R*)-5-hydroxy-2,4-dimethylpentyl acetate (98% ee) was synthesized according to the literature. (*45*)

(2*S*,4*R*)-5-{[tert-butyl(diphenyl)silyl]oxy}-2,4-dimethylpentyl acetate

To a solution of (2*S*,4*R*)-5-hydroxy-2,4-dimethylpentyl acetate (1.3 g, 7.5 mmol) in DMF (6 mL) were added TBDPSCl (2.4 mL, 9.4 mmol) and imidazole (1.0 g, 15 mmol) at rt. After 7 h the reaction mixture was diluted with EtOAc, washed with H_2_O, 2% HCl aq. and brine, and dried over Na_2_SO_4_. After filtration, the filtrate was concentrated in vacuo. The obtained residue was purified by chromatography on SiO_2_ to give (2*S*,4*R*)-5-{[tert-butyl(diphenyl)silyl]oxy}-2,4-dimethylpentyl acetate (2.97 g, 96%).

[α]^27^_D_ = +7.9 (98% ee, c 1.01, CHCl_3_)

^1^H NMR (400 MHz,CDCl_3_): δ = 7.68-7.65 (m, 4H), 7.44-7.35 (m, 6H), 3.93 (dd, *J* = 10.6, 5.4 Hz, 1H), 3.8 (dd, *J* = 10.6, 6.6 Hz, 1H), 3.48 (dd, *J* = 9.8, 5.4 Hz, 1H), 3.43 (dd, *J* = 10.0, 6.0 Hz, 1H), 2.03 (s, 3H), 1.83 (sex, *J* = 6.8 Hz, 1H), 1.74 (sex, *J* = 6.4 Hz, 1H), 1.54-1.48 (m, 1H), 1.05 (s, 9H), 0.98-0.90 (m, 1H), 0.95 (d, *J* = 6.4 Hz, 3H), 0.89 (d, *J* = 6.4 Hz, 3H).

^13^C NMR (100MHz, CDCl_3_); δ = 171.2, 135.6, 133.9, 129.5, 127.6, 69.3, 68.5, 37.3, 33.0, 30.0, 26.9, 20.9, 19.3, 17.6, 17.6.

(2*S*,4*R*)-5-{[tert-butyl(diphenyl)silyl]oxy}-2,4-dimethylpentan-1-ol

(2*S*,4*R*)-5-{[tert-Butyl(diphenyl)silyl]oxy}-2,4-dimethylpentyl acetate (200 mg, 0.48 mmol) was dissolved in MeOH (3.5 mL). To the solution was added K_2_CO_3_ (0.66g, 4.8 mmol) at rt. After 20 min the reaction was quenched by addition of a phosphate buffer (pH 7) and extracted with CH_2_Cl_2_ thrice. The combined organic layer was dried over Na_2_SO_4_. After filtration, the filtrate was concentrated in vacuo to give (2*S*,4*R*)-5-{[tert-butyl(diphenyl)silyl]oxy}-2,4-dimethylpentan-1-ol (175 mg, 97%).

[α]^28^_D_ = +0.5 (98% ee, c 1.20, CHCl_3_)

^1^H NMR (400 MHz,CDCl_3_): δ = 7.71-7.62 (m, 4H), 7.45-7.33 (m, 6H), 3.52-3.44 (m, 1H), 3.51 (dd, *J* = 9.8, 5.4 Hz, 1H), 3.42 (dd, *J* = 10.0, 6.4 Hz, 1H), 3.39-3.31 (m, 1H), 1.74 (sex, *J* = 6.4 Hz, 1H), 1.63 (sex, *J* = 6.5 Hz, 1H), 1.46 (quin, *J* = 6.8 Hz, 1H), 1.29-1.18 (m, 1H), 1.06 (s, 9H), 0.95 (d, *J* = 6.8 Hz, 3H), 0.94-0.87 (m, 1H), 0.89 (d, *J* = 6.8 Hz, 3H).

(2*S*,4*R*)-5-{[tert-butyl(diphenyl)silyl]oxy}-2,4-dimethylpentyl 4-methylbenzene-1-sulfonate

To a solution of (2*S*,4*R*)-5-{[tert-butyl(diphenyl)silyl]oxy}-2,4-dimethylpentan-1-ol (2.36 g, 6.4 mmol) in pyridine (25 mL) was added tosyl chloride (1.8 g, 9.6 mmol) at 0 °C. After 1 h the temperature was raised to rt. After 6 h the reaction was quenched by addition of H_2_O and 25% HCl aq. The mixture was extracted with EtOAc thrice and the combined organic layer was washed with 5% HCl aq. twice and sat. NaHCO_3_ aq. The organic layer was dried over Na_2_SO_4_. After filtration, the filtrate was azeotropically dried with benzene three times to give (2*S*,4*R*)-5-{[tert-butyl(diphenyl)silyl]oxy}-2,4-dimethylpentyl 4-methylbenzene-1-sulfonate (2.72 g, 81%).

[α]^27^_D_ = +4.8 (98% ee, c 0.91, CHCl_3_)

^1^H NMR (400 MHz,CDCl_3_): δ = 7.77 (d, *J* = 8,4 Hz, 2H), 7.69-7.59 (m, 4H), 7.47-7.34 (m, 6H), 7.31 (d, *J* = 7.6 Hz, 2H), 3.87 (dd, *J* = 9.4, 5.0 Hz, 1H), 3.72, (dd, *J* = 9.2, 6.8 Hz, 1H), 3.44 (dd, *J* = 10.0, 5.2 Hz, 1H), 3.37 (dd, *J* = 10.0, 6.0 Hz, 1H), 2.42 (s, 3H), 1.82 (sex, *J* = 6.6 Hz, 1H), 1.63 (sex, *J* = 6.4 Hz, 1H), 1.39 (quin, *J* = 6.8 Hz, 1H), 1.03 (s, 9H), 0.99-0.87 (m, 1H), 0.88 (d, *J* = 6.4 Hz, 3H), 0.85 (d, *J* = 6.8 Hz, 3H).

^13^C NMR (100MHz, CDCl_3_); δ = 144.5, 135.5, 133.8, 133.1, 129.7, 129.6, 127.8, 127.6, 75.1, 68.4, 36.8, 32.8, 30.3, 26.9, 21.6, 19.2, 17.5, 17.2.

*tert*-butyl{[(2*R*,4*R*)-2,4-dimethyloctyl]oxy}diphenylsilane

To a solution of *n*-propyl magnesium bromide (1.05 M in THF, 3.6 mL, 3.8 mmol) were added a solution of (2*S*,4*R*)-5-{[tert-butyl(diphenyl)silyl]oxy}-2,4-dimethylpentyl 4-methylbenzene-1-sulfonate (500 mg, 0.95 mmol) in THF (1 mL) and Li_2_CuCl_4_ (0.1 M in THF, 95 μL, 1 mol%) at –78 °C. The reaction was warmed to 0 °C and stirred for 19 h. Then the reaction was quenched by addition of sat. NH_4_Cl aq. The mixture was extracted with EtOAc thrice. The combined organic layer was dried over MgSO_4_. After filtration, the filtrate was concentrated in vacuo. The obtained residue was purified by chromatography on SiO_2_ to give *tert*-butyl{[(2*R*,4*R*)-2,4-dimethyloctyl]oxy}diphenylsilane (263 mg, 70%).

[α]^27^_D_ = +4.1 (98% ee, c 1.13, CHCl_3_)

^1^H NMR (400 MHz,CDCl_3_): δ = 7.71-7.63 (m, 4H), 7.46-7.32 (m, 6H), 3.51 (dd, *J* = 9.6, 5.2 Hz, 1H), 3.41 (dd, *J* = 9.6, 6.6 Hz, 1H), 1.80-1.66 (m, 1H), 1.49-1.32 (m, 2H), 1.31-1.13 (m, 6H), 1.06 (s, 9H), 0.92 (d, *J* = 6.8 Hz, 3H), 0.91-0.83 (m, 4H), 0.81 (d, *J* = 6.4 Hz, 3H).

^13^C NMR (100MHz, CDCl_3_); δ = 135.6, 134.1, 129.5, 127.5, 68.9, 41.2, 36.6, 33.2, 30.1, 29.1, 26.9, 23.1, 20.3, 19.3, 17.8, 14.2.

(2*R*,4*R*)-2,4-dimethyloctan-1-ol

To a solution of *tert*-butyl{[(2*R*,4*R*)-2,4-dimethyloctyl]oxy}diphenylsilane (500 mg, 1.26 mmol) in THF (5 mL) was added TBAF (1M in THF, 2.5 mL, 2.5 mmol) at rt. After 2.5 h the reaction was quenched by addition of sat. NH_4_Cl aq. The mixture was extracted with EtOAc thrice. The combined organic layer was washed with water and brine, dried over Na_2_SO_4_. After filtration, the filtrate was concentrated in vacuo. The obtained residue was purified by chromatography on SiO_2_ to give (2*R*,4*R*)-2,4-dimethyloctan-1-ol (130 mg, 65%).

[α]^27^_D_ = +9.7 (98% ee, c 1.05, CHCl_3_)

^1^H NMR (400 MHz,CDCl_3_): δ = 3.56-3.48 (m, 1H), 3.43-3.33 (m, 1H), 1.79-1.65 (m, 1H), 1.54-1.42 (m, 1H), 1.37-1.14 (m, 7H), 1.10-0.99 (m, 1H), 0.98-0.88 (m, 4H), 0.92 (d, *J* = 6.8 Hz, 3H), 0.88 (d, *J* = 6.4 Hz, 3H).

^13^C NMR (100MHz, CDCl_3_); δ = 68.4, 41.0, 36.3, 33.1, 30.0, 29.1, 23.0, 20.4, 17.3, 14.1.

5-{[(2*R*,4*R*)-2,4-dimethyloctyl]sulfanyl}-1-phenyl-1H-tetrazole

(2*R*,4*R*)-2,4-Dimethyloctan-1-ol (101 mg, 0.64 mmol), 1-phenyl-1*H*-tetrazole-5-thiol (137 mg, 0.77 mmol) and PPh_3_ (201 mg, 0.77 mmol) were dissolved in THF (2 mL). Then powder MS4A (70 mg) was added. After the suspension was stirred for 30 min at rt, the mixture was cooled to 0 °C and diethyl azodicarboxylate (40% in toluene, 360 μL, 0.77 mmol) was added. After stirred for 4 h at rt, the reaction was quenched by addition of water and sat. NH_4_Cl aq. The mixture was extracted with EtOAc thrice. The combined organic layer was washed with water and brine, dried over Na_2_SO_4_. After filtration, the filtrate was concentrated in vacuo. The obtained residue was purified by chromatography on SiO_2_ to give 5-{[(2*R*,4*R*)-2,4-dimethyloctyl]sulfanyl}-1-phenyl-1H-tetrazole (156 mg, 76%).

[α]^27^_D_ = -8.4 (98% ee, c 1.11, CHCl_3_)

^1^H NMR (400 MHz,CDCl_3_): δ = 7.62-7.50 (m, 5H), 3.50 (dd, *J* = 12.6, 5.0 Hz, 1H), 3.21 (dd, *J* = 12.4, 7.6 Hz, 1H), 2.10-1.97 (m, 1H), 1.56-1.49 (m, 1H), 1.44-1.37 (m, 1H), 1.34-1.14 (m, 6H), 1.14-1.02 (m, 1H), 1.03 (d, *J* = 6.8 Hz, 3H), 0.89 (d, *J* = 6.4 Hz, 3H), 0.87 (t, *J* = 6.8 Hz, 3H).

^13^C NMR (100MHz, CDCl_3_); δ = 154.7, 133.7, 130.0, 129.7, 123.8, 43.8, 40.5, 36.2, 30.4, 30.0, 29.0, 22.9, 20.1, 19.8, 14.1.

5-[(2*R*,4*R*)-2,4-dimethyloctane-1-sulfonyl]-1-phenyl-1H-tetrazole

5-{[(2*R*,4*R*)-2,4-Dimethyloctyl]sulfanyl}-1-phenyl-1H-tetrazole (150 mg, 0.47 mmol) was dissolved in CH_2_Cl_2_ (2.5 mL). The solution was cooled to 0 °C and then mCPBA (0.4 g, 2.35 mmol) was added. The reaction was stirred for 96 h at rt, then quenched by addition of 10% Na_2_S_2_O_3_ aq. and sat. Na_2_SO_3_. The mixture was extracted with CH_2_Cl_2_ thrice. The combined organic layer was washed with 10% Na_2_S_2_O_3_ aq., sat. Na_2_SO_3_ (twice) and brine. The organic layer was dried over Na_2_SO_4_. After filtration, the filtrate was concentrated in vacuo. The obtained residue was purified by chromatography on SiO_2_ to give 5-[(2*R*,4*R*)-2,4-dimethyloctane-1-sulfonyl]-1-phenyl-1H-tetrazole (94 mg, 57%).

[α]^27^_D_ = -0.1 (98% ee, c 0.94, CHCl_3_)

^1^H NMR (400 MHz,CDCl_3_): δ = 7.73-7.56 (m, 5H), 3.81 (dd, *J* = 14.4, 4.0 Hz, 1H), 3.53 (dd, *J* = 14.4, 8.4 Hz, 1H), 2.50-2.35 (m, 1H), 1.52-1.41 (m, 2H), 1.36-1.16 (m, 6H), 1.16 (d, *J* = 6.8 Hz, 3H), 1.13-1.03 (m, 1H), 0.88 (t, *J* = 6.8 Hz, 3H), 0.88 (d, *J* = 6.4 Hz, 3H).

^13^C NMR (100MHz, CDCl_3_); δ = 154.1, 133.0, 131.4, 129.6, 125.1, 61.8, 44.5, 35.9, 29.7, 28.9, 25.9, 22.9, 20.4, 19.8, 14.1.

*tert*-butyl{[(2*R*,4*R*)-2,4-dimethyldecyl]oxy}diphenylsilane

To a solution of *n*-pentyl magnesium bromide (2 M in Et_2_O, 3.2 mL, 6.4 mmol) were added a solution of (2*S*,4*R*)-5-{[tert-butyl(diphenyl)silyl]oxy}-2,4-dimethylpentyl 4-methylbenzene-1-sulfonate (847 mg, 1.6 mmol) in THF (1 mL) and Li_2_CuCl_4_ (0.05 M in THF, 320 μL, 1 mol%) at –78 °C. After 1h the reaction was warmed to 0 °C and stirred for another 18 h. Then the reaction was quenched by addition of sat. NH_4_Cl aq. The mixture was extracted with EtOAc thrice. The combined organic layer was dried over MgSO_4_. After filtration, the filtrate was concentrated in vacuo. The obtained residue was purified by chromatography on SiO_2_ to give *tert*-butyl{[(2*R*,4*R*)-2,4-dimethyldecyl]oxy}diphenylsilane (409 mg, 60%).

[α]^27^_D_ = +4.2 (98% ee, c 1.02, CHCl_3_)

^1^H NMR (400 MHz,CDCl_3_): δ = 7.70-7.63 (m, 4H), 7.45-7.33 (m, 6H), 3.50 (dd, *J* = 9.8, 5.4 Hz, 1H), 3.40 (dd, *J* = 9.8, 6.6 Hz, 1H), 1.80-1.65 (m, 1H), 1.48-1.31 (m, 2H), 1.31-1.14(m, 10H), 1.05 (s, 9H), 0.92 (d, *J* = 6.4 Hz, 3H), 0.91-0.83 (m, 4H), 0.81 (d, *J* = 6.4 Hz, 3H).

^13^C NMR (100MHz, CDCl_3_); δ = 135.6, 134.2, 129.4, 127.5, 69.0, 41.2, 36.9, 33.2, 32.0, 30.1, 29.7, 26.9, 26.8, 22.7, 20.3, 19.3, 17.8, 14.1.

(2*R*,4*R*)-2,4-dimethyldecan-1-ol

To a solution of *tert*-butyl{[(2*R*,4*R*)-2,4-dimethyldecyl]oxy}diphenylsilane (530 mg, 1.25 mmol) in THF (5 mL) was added TBAF (1M in THF, 2.5 mL, 2.5 mmol) at rt. After 4 h the reaction was quenched by addition of sat. NH_4_Cl aq. The mixture was extracted with EtOAc thrice. The combined organic layer was washed with water and brine, dried over Na_2_SO_4_. After filtration, the filtrate was concentrated in vacuo. The obtained residue was purified by chromatography on SiO_2_ to give (2*R*,4*R*)-2,4-dimethyldecan-1-ol (155 mg, 67%).

[α]^22^_D_ = +13 (98% ee, c 1.14, CHCl_3_)

^1^H NMR (400 MHz,CDCl_3_): δ = 3.57-3.48 (m, 1H), 3.43-3.34 (m, 1H), 1.79-1.65 (m, 1H), 1.53-1.42 (m, 1H), 1.38-1.14 (m, 11H), 1.12-0.99 (m, 1H), 0.98-0.87 (m, 4H), 0.92 (d, *J* = 6.4 Hz, 3H), 0.87 (d, *J* = 6.8 Hz, 3H).

^13^C NMR (100MHz, CDCl_3_); δ = 68.3, 41.0, 36.6, 33.1, 31.9, 30.0, 29.7, 26.8, 22.7, 20.3, 17.3, 14.1.

5-{[(2*R*,4*R*)-2,4-dimethyldecyl]sulfanyl}-1-phenyl-1H-tetrazole

(2*R*,4*R*)-2,4-Dimethyldecan-1-ol (155 mg, 0.83 mmol), 1-phenyl-1*H*-tetrazole-5-thiol (178 mg, 1.0 mmol) and PPh_3_ (261 mg, 1.0 mmol) were dissolved in THF (2.6 mL). Then powder MS4A (70 mg) was added. After the suspension was stirred for 30 min at rt, the mixture was cooled to 0 °C and diethyl azodicarboxylate (40% in toluene, 467 μL, 1.0 mmol) was added. After stirred for 4 h at rt, the reaction was quenched by addition of water and sat. NH_4_Cl aq. The mixture was extracted with EtOAc thrice. The combined organic layer was washed with water and brine, dried over Na_2_SO_4_. After filtration, the filtrate was concentrated in vacuo. The obtained residue was purified by chromatography on SiO_2_ to give 5-{[(2*R*,4*R*)-2,4-dimethyldecyl]sulfanyl}-1-phenyl-1H-tetrazole (259 mg, 90%).

[α]^25^_D_ = -8.2 (98% ee, c 1.18, CHCl_3_)

^1^H NMR (400 MHz,CDCl_3_): δ = 7.63–7.50 (m, 5H), 3.51 (dd, *J* = 12.4, 5.2 Hz, 1H), 3.22 (dd, *J* = 12.8, 8.0 Hz, 1H), 2.06–2.00 (m, 1H), 1.55-1.48 (m, 1H), 1.45-1.35 (m, 1H), 1.33–1.16 (m, 10H), 1.12–1.05 (m, 1H), 1.02 (d, *J* = 6.8 Hz, 3H), 0.89 (d, *J* = 6.4 Hz, 3H), 0.87 (t, *J* = 7.2 Hz, 3H).

^13^C NMR (100MHz, CDCl_3_); δ = 154.6, 133.7, 129.9, 129.7, 123.7, 43.8, 40.4, 36.5, 31.8, 30.3, 29.9, 29.5, 26.6, 22.6, 20.0, 19.7, 14.0.

5-[(2*R*,4*R*)-2,4-dimethyldecane-1-sulfonyl]-1-phenyl-1H-tetrazole

5-{[(2*R*,4*R*)-2,4-dimethyldecyl]sulfanyl}-1-phenyl-1H-tetrazole (253 mg, 0.73 mmol) was dissolved in CH_2_Cl_2_ (4 mL). The solution was cooled to 0 °C and then mCPBA (0.63 g, 3.65 mmol) was added. The reaction was stirred for 64 h at rt, then quenched by addition of 10% Na_2_S_2_O_3_ aq. and sat. Na_2_SO_3_. The mixture was extracted with CH_2_Cl_2_ thrice. The combined organic layer was washed with 10% Na_2_S_2_O_3_ aq., sat. Na_2_SO_3_ (twice) and brine. The organic layer was dried over Na_2_SO_4_. After filtration, the filtrate was concentrated in vacuo. The obtained residue was purified by chromatography on SiO_2_ to give 5-[(2*R*,4*R*)-2,4-dimethyldecane-1-sulfonyl]-1-phenyl-1H-tetrazole (128 mg, 46%).

[α]^24^D = -0.1 (98% ee, c 1.07, CHCl_3_)

^1^H NMR (400 MHz,CDCl_3_): δ = 7.72-7.57 (m, 5H), 3.81 (dd, *J* = 14.4, 4.0 Hz, 1H), 3.53 (dd, *J* = 14.2, 8.2 Hz, 1H), 2.50-2.35 (m, 1H), 1.54-1.40 (m, 2H), 1.36-1.13 (m, 10H), 1.16 (d, *J* = 6.8 Hz, 3H), 1.12-1.01 (m, 1H), 0.88 (d, *J* = 6.4 Hz, 3H), 0.88 (t, *J* = 6.8 Hz, 3H).

^13^C NMR (100MHz, CDCl_3_); δ = 154.0, 133.0, 131.4, 129.6, 125.1, 61.7, 44.5, 36.3, 31.8, 29.7, 29.5, 26.6, 25.8, 22.6, 20.4, 19.8, 14.1.

(4*S*)-4-methylheptadecanal

Mg turning (1.48 g, 60 mmol) was placed in a flask. Then THF (11 mL) and 1,2-dibromoethane (157 μL, 1.8 mmol) were added in this order to activate Mg. Then a solution of 1-bromoundecane (10 mL, 45.1 mmol) in THF (22 mL) was added dropwise (not very exothermic), and the mixture was refluxed for 30 min. After cooled to –78 °C, THF (21 mL), a solution of (3*R*)-3,7-dimethyloct-6-en-1-yl 4-methylbenzene-1-sulfonate (3.5 g, 11.3 mmol) in THF (6.5 mL), and Li_2_CuCl_4_ (0.1 M in THF, 1.1 mL, 1 mol%) were added. The reaction was allowed to warm to rt. After 15 h the reaction was quenched by addition of sat. NH_4_Cl aq. The mixture was extracted with hexane thrice. The combined organic layer was dried over MgSO_4_. After filtration, the filtrate was concentrated in vacuo. The obtained residue was first purified by chromatography on SiO_2_ using hexane as an eluent. The obtained mixture with low polarity was further purified by Kugelrohr-distillation (70 °C, 100 Pa). The remaining residue was almost pure desired intermediate, (6*S*)-2,6-dimethylnonadec-2-ene. The obtained alkene was dissolved in CH_2_Cl_2_ (180 mL). Then NaHCO_3_ (958 mg, 11.4 mmol), hexane (110 mL) and MeOH (20 mL) were added. The reaction was cooled to –78 °C. Ozone was bubbled through the solution at –78 °C until the reaction took on persistent blue color (2.5 h). Then O_2_ and argon were bubbled in this order to expel O_3_ and O_2_, respectively. Me_2_S (12.6 mL, 171 mmol) was added at –78 °C and the reaction was allowed to warm to rt. After 14 h H_2_O was added and the mixture was extracted with CH_2_Cl_2_ thrice. The combined organic layer was dried over MgSO_4_. After filtration, the filtrate was concentrated in vacuo. The obtained residue was purified by chromatography on SiO_2_ to give (4*S*)-4-methylheptadecanal (1.32 g, 43% in two steps).

[α]^28^_D_ = +2.7(c 1.00, CHCl_3_)

^1^H NMR (400 MHz,CDCl_3_): δ = 9.77 (t, *J* = 1.6 Hz, 1H), 2.45-2.39 (m, 2H), 1.70-1.61 (m, 1H), 1.48-1.38 (m, 2H), 1.3-1.26 (m, 23H), 1.16-1.09 (m, 1H), 0.87 (d, *J* = 3.2 Hz, 3H), 0.88 (t, *J* = 6.8 Hz, 3H).

^13^C NMR (100MHz, CDCl_3_); δ = 203.0, 41.7, 36.7, 32.3, 31.9, 29.9, 29.9, 29.7, 29.7, 29.7, 29.6 29.6, 29.3, 28.9, 26.9, 22.3, 19.3, 14.1.

(4*S*)-4-methylnonadecanal

Mg turning (0.41 g, 16.7 mmol) was placed in a flask. Then THF (3 mL) and 1,2-dibromoethane (43 μL, 0.5 mmol) were added in this order to activate Mg. Then a solution of 1-bromotridecane (3.26 g, 12.4 mmol) in THF (6.2 mL) was added dropwise (not very exothermic), and the mixture was refluxed for 30 min. After cooled to –78 °C, THF (6 mL), a solution of (3*R*)-3,7-dimethyloct-6-en-1-yl 4-methylbenzene-1-sulfonate (0.96 g, 3.1 mmol) in THF (2 mL), and Li_2_CuCl_4_ (0.1 M in THF, 0.31 mL, 1 mol%) were added. The reaction was allowed to warm to rt. After 22 h the reaction was quenched by addition of sat. NH_4_Cl aq. The mixture was extracted with hexane thrice. The combined organic layer was dried over MgSO_4_. After filtration, the filtrate was concentrated in vacuo. The obtained residue was first purified by chromatography on SiO_2_ using hexane as an eluent. The obtained mixture with low polarity was further purified by Kugelrohr-distillation (85 °C, 50 Pa). The remaining residue was almost pure desired intermediate, (6*S*)-2,6-dimethylhenicos-2-ene. The obtained alkene was dissolved in CH_2_Cl_2_ (60 mL). Then NaHCO_3_ (302 mg, 3.6 mmol), hexane (35 mL) and MeOH (6 mL) were added. The reaction was cooled to –78 °C. Ozone was bubbled through the solution at –78 °C until the reaction took on persistent blue color (1 h). Then O_2_ and argon were bubbled in this order to expel O_3_ and O_2_, respectively. Me_2_S (4 mL, 54 mmol) was added at –78 °C and the reaction was allowed to warm to rt. After 15 h H_2_O was added and the mixture was extracted with CH_2_Cl_2_ thrice. The combined organic layer was dried over MgSO_4_. After filtration, the filtrate was concentrated in vacuo. The obtained residue was purified by chromatography on SiO_2_ to give (4*S*)-4-methylnonadecanal (0.59 g, 55% in two steps).

[α]^28^_D_ = -0.5(c 0.88, CHCl_3_)

^1^H NMR (400 MHz,CDCl_3_): δ = 9.77 (t, *J* = 1.6 Hz, 1H), 2.48-2.37 (m, 2H), 1.69-1.61 (m, 1H), 1.48-1.38 (m, 2H), 1.33-1.21 (m, 27H), 1.12-1.09 (m, 1H), 0.87 (d, *J* = 3.2 Hz, 3H), 0.88 (t, *J* = 6.8 Hz, 3H).

^13^C NMR (100MHz, CDCl_3_); δ = 203.0, 41.7, 36.7, 32.3, 31.9, 29.9, 29.9, 29.8, 29.7, 29.7, 29.7, 29.6, 29.6, 29.4, 29.3, 28.9, 26.9, 22.3, 19.3, 14.1.

(4*S*)-4-benzyl-3-pentadecanoyl-1,3-oxazolidin-2-one

To a solution of *n*-pentadecanoic acid (3.3 g, 13.6 mmol) in CH_2_Cl_2_ (130 mL) were added trimethylamine (4.4 mL, 31.6 mmol) and pivaloyl chloride (1.9 mL, 15.8 mmol) at rt. After 3 h (*S*)-4-benzyl-2-oxazolidinone (2 g, 11.3 mmol) and 4-dimethylaminopyridine (110 mg, 0.9 mmol) were added. The reaction was stirred at 45 °C for 21 h and then cooled to rt. Water was added and the reaction mixture was extracted with CH_2_Cl_2_ thrice. The combined organic layer was dried over Na_2_SO_4_. After filtration, the filtrate was concentrated in vacuo. The obtained residue was purified by chromatography on SiO_2_ and amine-functionalized SiO_2_ (WAKO gel^®^ 50NH_2_) to give (4*S*)-4-benzyl-3-pentadecanoyl-1,3-oxazolidin-2-one (3.7 g, 68%).

[α]^24^_D_ = +38.4 (c 1.16, CHCl_3_)

^1^H NMR (400 MHz,CDCl_3_): δ = 7.35-7.20 (m, 5H), 4.70-4.64 (m, 1H), 4.22-4.15 (m, 2H), 3.30 (dd, *J* = 13.2, 3.2 Hz, 1H), 3.01-2.85 (m, 2H), 2.76 (dd, *J* = 13.2, 9.6 Hz, 1H), 1.71-1.65 (m, 2H), 1.43-1.18 (m, 22H), 0.8 (t, *J* = 6.8 Hz, 3H).

(4*S*)-4-benzyl-3-[(2*S*)-2-methylpentadecanoyl]-1,3-oxazolidin-2-one

(4*S*)-4-Benzyl-3-pentadecanoyl-1,3-oxazolidin-2-one (3.5 g, 8.7 mmol) was dissolved in THF (87 mL) and powder MS4A (1.5 g) was added. After 30 min the reaction was cooled to –78 °C. NaHMDS (1 M in THF, 17.4 mL, 17.4 mmol) was added to the reaction and the mixture was stirred at –78 °C for 30 min. Then MeI (5.4 mL, 87 mmol) was added and the reaction was stirred at –40 °C for 1.5 h. The reaction was quenched by addition of water and the mixture was extracted with EtOAc thrice. The combined organic layer was dried over MgSO_4_. After filtration, the filtrate was concentrated in vacuo. The obtained residue was purified by chromatography on SiO_2_ to give (4*S*)-4-benzyl-3-[(2*S*)-2-methylpentadecanoyl]-1,3-oxazolidin-2-one (3.1 g, 86%).

[α]^27^_D_ = +52.5 (c 0.92, CHCl_3_)

^1^H NMR (400 MHz,CDCl_3_): δ = 7.35-7.21 (m, 5H), 4.70-4.65 (m, 1H), 4.22-4.15 (m, 2H), 3.73-3.67 (m, 1H), 3.27 (dd, *J* = 13.2, 3.2 Hz, 1H), 2.76 (dd, *J* = 13.2, 9.6 Hz, 1H), 1.80-1.66 (m, 2H), 1.47-1.24 (m, 22H), 1.23 (t, *J* = 6.8 Hz, 3H), 0.88 (t, *J* = 6.8 Hz, 3H).

(2*S*)-2-methylpentadecan-1-ol

(4*S*)-4-Benzyl-3-[(2*S*)-2-methylpentadecanoyl]-1,3-oxazolidin-2-one (1.5 g, 3.6 mmol) was dissolved in Et_2_O (60 mL). The solution was cooled to –10 °C, then EtOH (0.21 mL, 4.3 mmol) and LiBH_4_ (85 mg, 4.3 mmol) were added. After 1.5 h 1 M NaOH aq. (20 mL) was added and the reaction was allowed to warm to 0 °C. After 15 min Et_2_O and brine were added. The mixture was extracted with Et_2_O thrice. The combined organic layer was dried over MgSO_4_. After filtration, the filtrate was concentrated in vacuo. The obtained residue was purified by chromatography on SiO_2_ to give (2*S*)-2-methylpentadecan-1-ol (796 mg, 91%).

[α]^24^_D_ = -9.2 (c 1.34, CHCl_3_)

^1^H NMR (400 MHz,CDCl_3_): δ = 3.54-3.48 (m, 1H), 3.44-3.39 (m, 1H), 1.65-1.58 (m, 1H), 1.43-1.23 (m, 24H), 1.13-1.08 (m, 1H), 0.91 (d, *J* = 6.4 Hz, 3H), 0,88 (t, *J* = 6.8 Hz, 3H).

(2*S*)-2-methylpentadecanal

To a solution of oxalyl chloride (0.15 mL, 1.66 mmol) in CH_2_Cl_2_ (5.5 mL) was added DMSO (0.28 mL, 3.32 mmol) dropwise at –78 °C. After 30 min (2*S*)-2-methylpentadecan-1-ol (200 mg, 0.83 mmol) was added. After 2.5 h triethylamine (0.7 mL, 4.98 mmol) was added slowly, then the reaction was allowed to warm to rt. After 18 h sat. NH_4_Cl aq. was added, then the mixture was extracted with CH_2_Cl_2_ twice. The combined organic layer was dried over MgSO_4_. After filtration, the filtrate was concentrated in vacuo. The obtained residue was purified by chromatography on SiO_2_ to give (2*S*)-2-methylpentadecanal (97 mg, 49%).

(5*R*,7*S*,12*S*)-5,7,12-trimethylpentacosane

5-[(2*R*,4*R*)-2,4-Dimethyloctane-1-sulfonyl]-1-phenyl-1H-tetrazole (116 mg, 0.33 mmol) was dissolved in THF (2.5 mL) and powder MS4A (100 mg) was added. After 30 min the reaction was cooled to –78 °C. LiHMDS (0.25 M in THF, 1.44 mL, 0.36 mmol) was then added to the solution, followed by addition of (4*S*)-4-methylheptadecanal (134 mg, 0.5 mmol). After 3 h the reaction was allowed to warm to rt, then stirred for 16 h. Sat. NH_4_Cl aq. was added and the mixture was extracted with Et_2_O thrice. The combined organic layer was dried over MgSO_4_. After filtration, the filtrate was concentrated in vacuo. The obtained residue was purified by chromatography on SiO_2_ to give (5*R*,7*R*,12*S*)-5,7,12-trimethylpentacos-8-ene. The obtained alkene was dissolved in EtOAc (10 mL). Palladium-activated carbon (WAKO, 10% Pd, 40 mg, 11 mol%) was added and the atmosphere was exchanged to H_2_. The H_2_ atmosphere was kept using a balloon. The reaction was stirred under 6 h. The mixture was filtered on celite and the filtrate was concentrated in vacuo to give (5*R*,7*S*,12*S*)-5,7,12-trimethylpentacosane (101.2 mg, 78% in two steps).

[α]^28^D = –6.8 (98% ee, c 0.20, CHCl_3_)

^1^H NMR (400 MHz,CDCl_3_): δ = 1.49-1.43 (m, 4H), 1.35-1.16 (m, 33H), 1.08-0.99 (m, 6H), 0.94-0.80 (m, 15H).

^13^C NMR (100MHz, CDCl_3_); δ = 45.2, 37.1, 37.0, 36.9, 36.6, 32.8, 32.0, 30.0, 30.0, 29.9, 29.7, 29.7, 29.6, 29.4, 29.3, 29.2, 27.4, 27.3, 27.1, 23.1, 23.0, 22.7, 20.3, 20.3, 19.8, 19.6, 14.2, 14.1.

MS (m/z, abundance): 337 (6, M-57), 295 (4), 267 (10), 225 (2), 211 (6), 183 (8), 141 (10), 127 (30), 99 (28), 85 (88), 71 (100).

(5*R*,7*S*,12*S*)-5,7,12-trimethylheptacosane

5-[(2*R*,4*R*)-2,4-Dimethyloctane-1-sulfonyl]-1-phenyl-1H-tetrazole (70 mg, 0.2 mmol) was dissolved in THF (2 mL) and powder MS4A (70 mg) was added. After 30 min the reaction was cooled to –78 °C. LiHMDS (0.25 M in THF, 0.88 mL, 0.22 mmol) was then added to the solution, followed by addition of (4*S*)-4-methylnonadecanal (89 mg, 0.3 mmol). After 3 h the reaction was allowed to warm to rt, then stirred for 18 h. Sat. NH_4_Cl aq. was added and the mixture was extracted with Et_2_O thrice. The combined organic layer was dried over MgSO_4_. After filtration, the filtrate was concentrated in vacuo. The obtained residue was purified by chromatography on SiO_2_ to give (5*R*,7*R*,12*S*)-5,7,12-trimethylheptacos-8-ene. The obtained alkene was dissolved in EtOAc (5 mL). Palladium-activated carbon (WAKO, 10% Pd, 20 mg, 9 mol%) was added and the atmosphere was exchanged to H_2_. The H_2_ atmosphere was kept using a balloon. The reaction was stirred under 2 h. The mixture was filtered on celite and the filtrate was concentrated in vacuo to give (5*R*,7*S*,12*S*)-5,7,12-trimethylheptacosane (48.3 mg, 58% in two steps).

[α]^27^_D =_ –4.5 (98% ee, c 0.78, CHCl_3_)

^1^H NMR (400 MHz,CDCl_3_): δ = 1.52-1.41 (m, 4H), 1.38-1.13 (m, 37H), 1.12-0.97 (m, 6H), 0.96-0.77 (m, 15H).

^13^C NMR (100MHz, CDCl_3_); δ = 45.2, 37.2, 37.1, 36.9, 36.6, 32.8, 32.0, 30.1, 30.0, 30.0, 29.8, 29.7, 29.7, 29.4, 29.4, 29.2, 27.5, 27.3, 27.1, 23.1, 23.1, 22.7, 20.3, 20.3, 19.8, 19.7, 14.2, 14.1.

MS (m/z, abundance): 407 (2, M-15), 365 (15), 337 (7), 323 (14), 295 (24), 207 (14), 183 (16), 169 (10), 141 (18), 127 (58), 113 (28), 99 (38), 85 (100), 71 (96).

(7*R*,9*S*,12*S*)-7,9,12-trimethylpentacosane

5-[(2*R*,4*R*)-2,4-dimethyldecane-1-sulfonyl]-1-phenyl-1H-tetrazole (102 mg, 0.27 mmol) was dissolved in THF (1 mL) and powder MS4A (80 mg) was added. After 30 min the reaction was cooled to –78 °C. LiHMDS (0.25 M in THF, 1.2 mL, 0.30 mmol) was then added to the solution, followed by addition of (2*S*)-2-methylpentadecanal (97 mg, 0.4 mmol). After 3 h the reaction was allowed to warm to rt, then stirred for 16 h. Sat. NH_4_Cl aq. was added and the mixture was extracted with Et_2_O thrice. The combined organic layer was dried over MgSO_4_. After filtration, the filtrate was concentrated in vacuo. The obtained residue was purified by chromatography on SiO_2_ to give (7*R*,9*R*,12*S*)-7,9,12-trimethylpentacos-10-ene. The obtained alkene was dissolved in EtOAc (4 mL). Palladium-activated carbon (WAKO, 10% Pd, 15 mg, 5 mol%) was added and the atmosphere was exchanged to H_2_. The H_2_ atmosphere was kept using a balloon. The reaction was stirred under 2 h. The mixture was filtered on celite and the filtrate was concentrated in vacuo to give (7*R*,9*S*,12*S*)-7,9,12-trimethylpentacosane (28 mg, 27% in two steps).

[α]^24^_D =_ –2.0 (98% ee, c 0.93, CHCl_3_)

^1^H NMR (400 MHz,CDCl_3_): δ = 1.53-1.37 (m, 4H), 1.35-1.14 (m, 34H), 1.13-0.95 (m, 5H), 0.94-0.77 (m, 15H).

^13^C NMR (100MHz, CDCl_3_); δ = 45.3, 45.1, 37.3, 37.0, 36.8, 34.2, 34.1, 33.1, 33.1, 32.0, 31.9, 30.3, 30.2, 30.1, 30.0, 29.8, 29.7, 29.7, 29.4, 27.1, 26.9, 22.7, 20.4, 20.4, 20.3, 19.9, 19.7, 14.1.

MS (m/z, abundance): 309 (4, M-85), 281 (5), 267 (4), 207 (16), 183 (18), 155 (17), 141 (7), 127 (11), 113 (14), 99 (23), 85 (56), 71 (86), 57 (100).

**References**

38. T.-S. Li, J.-T. Li, H.-Z. Li. Modified and convenient preparation of silica impregnated with silver nitrate and its application to the separation of steroids and triterpenes. *J. Chromatogr. A* **715**, 372-375 (1995).

39. H. Noguchi, T. Aoyama, T. Shioiri. Total synthesis of analogs of topostin B, A DNA topoisomerase I inhibitor. Part 1. Synthesis of fragments of topostin B-1 analogs. *Tetrahedron*. **51**, 10531-10544 (1995).

40. J. Belmar, R. L. Funk, Total Synthesis of (±)-Perophoramidine. *J. Am. Chem. Soc*. **134**, 16941-16943 (2012).

41. M. Buck, J. M. Chong, Alkylation of 1-alkynes in THF. *Tetrahedron Lett.* **42**, 5825-5827 (2001).

42. L. E. Overman, M. J. Brown, S. F. McCann, (Z)-4-(TRIMETHYLSILYL)-3-BUTEN-1-OL [3-Buten-1-ol, 4-(trimethylsilyl)-, (Z)-]. *Org. Synth.* **68**, 182- (1990).

43. C. T. Lohan, M. J. van Belkum, S. A. Cochrane, Z. Huang, C. S. Sit, L. M. McMullen, J. C. Vederas, Biochemical, structural, and genetic characterization of tridecaptin A₁, an antagonist of Campylobacter jejuni. *ChemBioChem* **15**, 243–249 (2014).

44. S. Chandrasekhar, S. S. Sultana, Stereoselective synthesis of the C1–C20 segment of the microsclerodermins A and B. *Tetrahedron Lett.* **47**, 7255–7258 (2016).

45. N. N. Doan, T. N. Le, H. C. Nguyen, P. E. Hansen, F. Duus, Ultrasound Assisted Synthesis of 5, 9-Dimethylpentadecane and 5, 9-Dimethylhexadecane–the Sex Pheromones of Leucoptera coffeella. *Molecules* **12**, 2080–2088 (2007).

46. C. R. Johnson, G. A. Dutra, Reactions of lithium diorganocuprates(I) with tosylates. II. Stereochemical, kinetic, and mechanistic aspects. *J. Am. Chem. Soc.* **95**, 7777–7782 (1973).

47. K. Fujita, K. Mori, Synthesis of (2R,4R)‐Supellapyrone, the Sex Pheromone of the Brownbanded Cockroach, Supella longipalpa, and Its Three Stereoisomers. *Eur. J. Org. Chem.* 493–502 (2012).
